# A review of biomimetic scaffolds for bone regeneration: Toward a cell‐free strategy

**DOI:** 10.1002/btm2.10206

**Published:** 2020-12-15

**Authors:** Sijing Jiang, Mohan Wang, Jiacai He

**Affiliations:** ^1^ Department of Plastic Surgery First Affiliated Hospital of Anhui Medical University, Anhui Medical University Hefei China; ^2^ Stomatologic Hospital & College, Anhui Medical University, Key Laboratory of Oral Diseases Research of Anhui Province Hefei China

**Keywords:** acellular, biomimetic, bone regeneration, scaffold, functionalization

## Abstract

In clinical terms, bone grafting currently involves the application of autogenous, allogeneic, or xenogeneic bone grafts, as well as natural or artificially synthesized materials, such as polymers, bioceramics, and other composites. Many of these are associated with limitations. The ideal scaffold for bone tissue engineering should provide mechanical support while promoting osteogenesis, osteoconduction, and even osteoinduction. There are various structural complications and engineering difficulties to be considered. Here, we describe the biomimetic possibilities of the modification of natural or synthetic materials through physical and chemical design to facilitate bone tissue repair. This review summarizes recent progresses in the strategies for constructing biomimetic scaffolds, including ion‐functionalized scaffolds, decellularized extracellular matrix scaffolds, and micro‐ and nano‐scale biomimetic scaffold structures, as well as reactive scaffolds induced by physical factors, and other acellular scaffolds. The fabrication techniques for these scaffolds, along with current strategies in clinical bone repair, are described. The developments in each category are discussed in terms of the connection between the scaffold materials and tissue repair, as well as the interactions with endogenous cells. As the advances in bone tissue engineering move toward application in the clinical setting, the demonstration of the therapeutic efficacy of these novel scaffold designs is critical.

## 1 INTRODUCTION

Despite the promising prospect of cell treatments for tissue engineering, the general application of engineered tissue and stem cells has not yet achieved clinical reality. Many details, including the selection, delivery, viability, and phenotypic stability of the cells, in addition to time‐consuming therapies, supervisory issues, and high costs, need to be optimized.^1^, ^2^, ^3^

In view of this, the field of acellular biomaterials is progressing and is becoming a practical alternative to cell‐based therapies. Previously, acellular materials only were regarded as fillers for the tissue defects,^4^ but now are able to be engineered into scaffolds that can interact with surrounding cells and tissues to alter the traditional recovery processes from disease or trauma.^3^ In this review, we address an acellular approach utilizing cell‐free biomaterials which can be modified through physical and chemical strategies and takes advantage of the capacity for tissue regeneration via interaction with local stem cells and surrounding tissues and which promises to avoid the scientific and regulatory disputes of cell‐based materials.

Mesenchymal stem cells (MSCs) have great potential in cell‐based treatments for tissue repair and regeneration and are used extensively, because of their proliferation, multilineage potential, immune regulatory, and anti‐inflammatory effects. However, as exogenous cells, there is insufficient understanding of the interplay between the cells and the implanted materials. There are also risks of supraphysiological dosages required to produce the necessary efficacy, potential side‐effects, and the challenges of achieving the ideal release kinetics to stimulate the surrounding cells. These factors indicate the necessity of using cell‐free materials. For acellular materials, it is important to focus on the characteristics of the designed scaffolds, as well as their biodegradability, porosity, biocompatibility, and, with reference to bone regeneration, their osteoconduction.

Natural bone is composed of complicated hierarchical architectures from nanoscale to macroscale, combining distinctive biological properties and high mechanical strength. The native bone matrix is composed of inorganic components (hydroxyapatite) and organic components (collagen‐I), which have been widely used in simulated biomimetics due to their outstanding osteoconductivity and biocompatibility.^5^, ^6^ Foreign ions (such as Zn^2+^, Sr^2+^, Si^4+^) could be doped in hydroxyapatite (HA) or another natural or polymeric material, even bioceramic material, that would effectively mimic the mineralization process of natural bone and hence promote osteoinduction and osteointegration.^7^, ^8^, ^9^, ^10^ Another biomimicry target is the extracellular matrix (ECM), a complex network of polysaccharides and proteins secreted and regulated by cells that provides biochemical signals for the modulation of cell activities and also as a bridge for connecting cells and materials.^11^, ^12^ Decellularized extracellular matrix (dECM) deposited on a biphasic calcium phosphate (BCP) scaffold was prepared through two different methods and was shown to be effective in promoting the bioactivity of scaffolds and providing an appropriate microenvironment for tissue regeneration, especially for osteogenesis.^13^ In addition, we consider literature describing the mimicry of native bone ECM for bone tissue engineering.^14^ This review focuses on the recreation of the chemical and physical cues within native ECM in relation to different aspects, aiming to apply this knowledge to the development of acellular materials for bone regeneration.

Additionally, the defined control of topological features of scaffold materials is dependent on ordered and elaborated preparation methods such as advanced three‐dimensional (3D) printing technology. Variations in surface roughness and fiber alignment, especially interconnected pore structures, could be prepared by 3D printing technology that is able to produce sophisticated architectures with 3D features.^15^, ^16^ By mimicking such micro‐/nanostructural characteristics of bone tissues, cell actions such as migration, adhesion, proliferation, as well as differentiation could be regulated, further promoting bone regeneration.^17^, ^18^ Meanwhile, in addition to biochemical signals, ambient physical stimuli such as electrical and magnetic factors, can also influence cells and are able to further prompt bone regeneration.^19^, ^20^, ^21^ Based on previous reports, bone tissue, which possesses piezoelectric properties, can generate charges or potentials in response to mechanical stimuli and have the capacity of enhancing bone growth.^22^ The application of magnetoelectric scaffolds and restoration of the physiological electric microenvironment in bone tissue regeneration can further regulate cell fate and optimize biomaterial design.^19^, ^23^ Evolving strategies that combine external environmental physical cues with the intrinsic features of materials and modulated scaffold systems can thus be utilized to synergistically drive bone regeneration. Moreover, mechanical parameters of materials or cells that can be controlled are important for regulating cellular fate.^24^, ^25^, ^26^ For example, 3D scaffolds coated with Ti surfaces can provide similar rigidity to cartilage (0.5–3 MPa), allowing cell growth.^27^ Hydrogels which possess flexible and tunable stress relaxation could guide cell behavior and fate. It has been found that cells cultured in gels with faster relaxation, spreading of MSCs was faster, as well as boosting both the proliferation and osteogenic differentiation of the cells.^25^ Innate growth factors can be stimulated by cellular adhesive forces, a feature that has been used by flexible aptamer technology to produce mimics of the transforming growth factor‐beta large latent complex. Traction forces can thus act as triggers activating specific biological responses and thus have potential applications in both biological research and regenerative medicine.^26^

Lastly, energy‐driven, photothermally modified, thermodynamically controlled, and photoluminescent biodegradable materials have been prepared by researchers.^28^, ^29^, ^30^ Tissue regeneration is dependent upon cellular bioenergetics (CBE) which, within bioenergetic‐active material (BAM) scaffolds, promote mitochondrial membrane potential (ΔΨm) to provide elevated bioenergetic levels and further accelerate bone repair.^28^ For the simultaneous treatment of osteosarcoma and tissue regeneration in clinical terms, an innovative multifunctional scaffold with temperature‐controlled characteristics has been reported which can efficiently eliminate human osteosarcoma cells at 48°C, while enhancing osteogenesis of BMSCs at a temperature of 42 ± 0.5°C using 808‐nm near‐infrared (NIR) light irradiation.^30^

**FIGURE 1:**
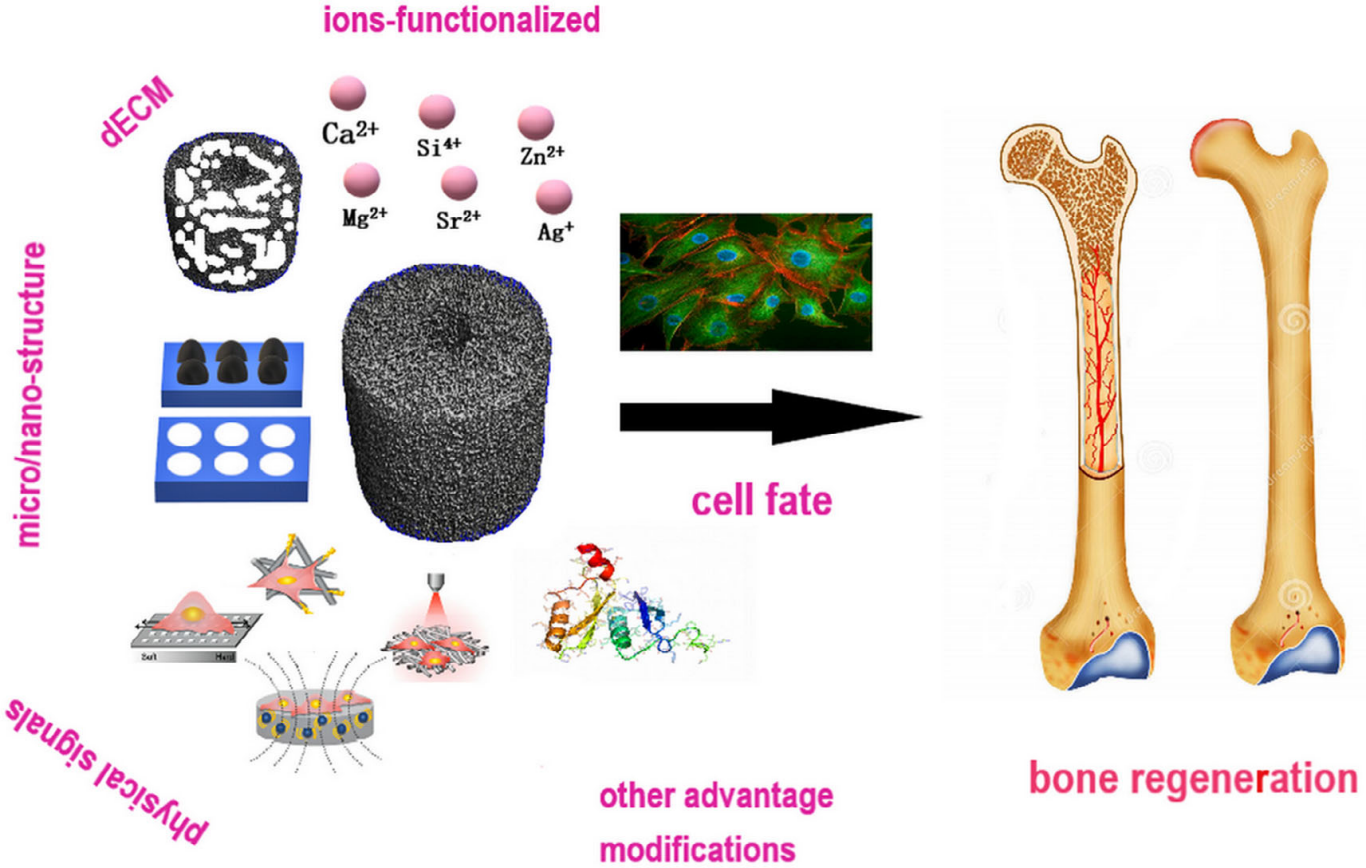
Schematic diagram of construction strategies for biomimetic bone scaffolds for cell guidance in bone regeneration

This review aims to describe the manufacture of biomimetic bone materials, including the different methods used, their structures, and scales from microscale to macroscale, to promote the physical and chemical modification of structural surface features to regulate bone growth. We describe the latest progress in biomimetic strategies, including ion doping, functionalization of the dECM, and ambient physical stimulation, from micro‐/nanoscale to macroscale, as well as the advantages of other functional scaffold materials (Figure 1). Cellular responses to these scaffolds in vitro, as well as the in vivo process of new bone formation produced by these strategies will be highlighted. This summary of recent advances in these fields identifies important issues and future directions for the design of biomimetic scaffold materials, specifically in terms of promoting cellular behavioral changes toward substrates in the process of bone tissue regeneration.^31^

## 2 BIOMIMETIC ION‐FUNCTIONALIZED SCAFFOLDS

**FIGURE 2:**
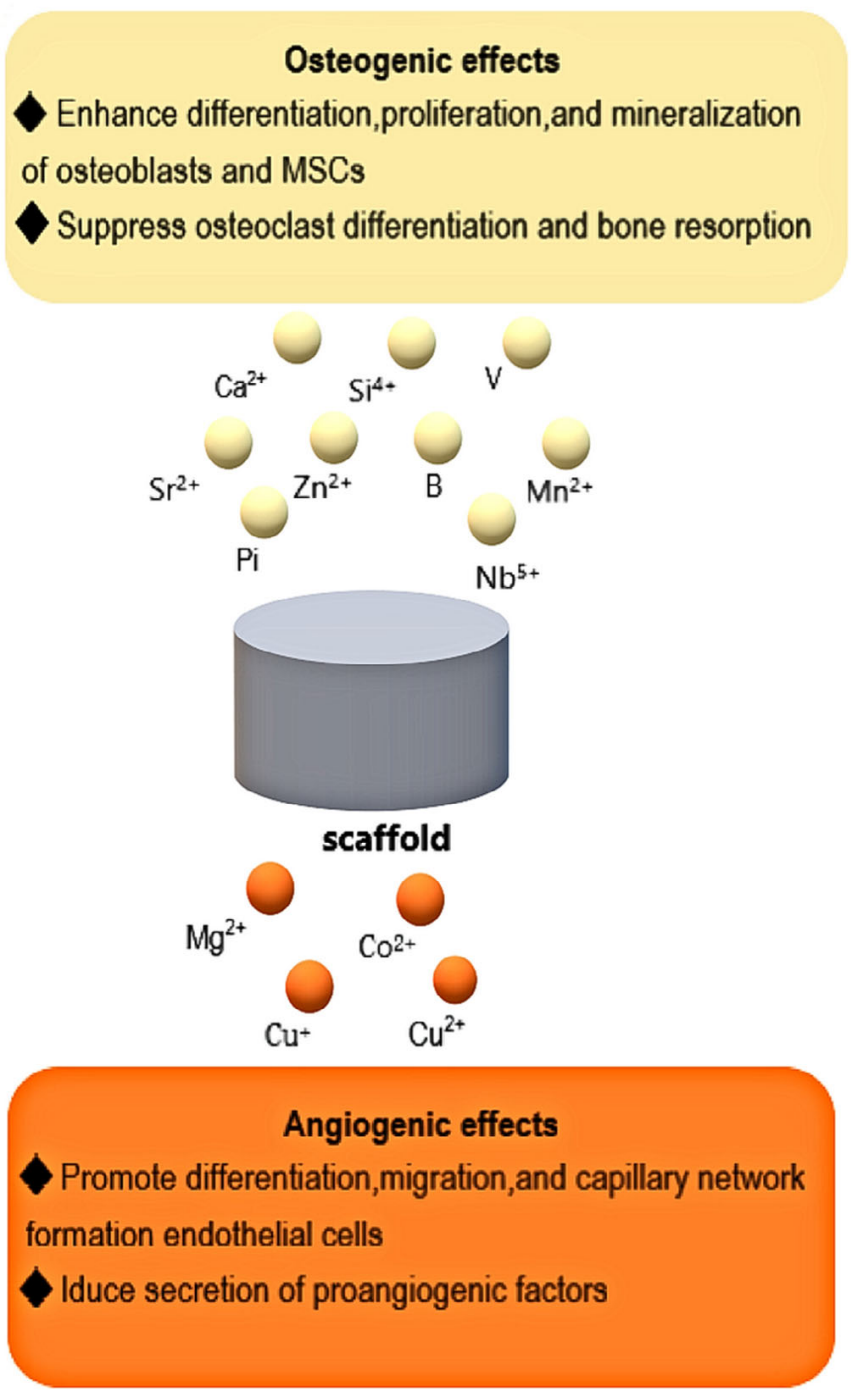
Schematic of inorganic ions released from a biomaterial scaffold and their associated therapeutic effects toward bone regeneration. MSC, mesenchymal stem cell

Several trace inorganic ions have been discovered that are conducive to bone tissue regeneration.^32^ This has inspired researchers to explore various bioactive glass dissolution products and doping strategies, as well as synthetic HA, bioactive glass, and other materials involving natural/polymer materials. In comparison with other cues of promoting osteogenesis, the superiority of applying inorganic ions to facilitate bone trauma repair is multifaceted, including cost‐efficiency, improved stability and simplicity, and outstanding efficacy at low concentrations.^33^, ^34^, ^35^ In this section, we will pay attention to ion‐doped and dissoluble scaffold materials with enhanced biological activities involving osteogenesis, angiogenesis and antibacterial properties that are involved in the application of these ion‐relevant materials. Figure 2 shows the distinctive therapeutic effects of these ions toward bone regeneration released from a biomaterial scaffold.

### 2.1 Ion‐doped materials

Neščáková et al. fabricated mesoporous bioactive glass nanoparticles (MBGNs) using the SiO_2_–CaO system with Zn^2+^ ions being doped in MBGNs. The Zn‐MBGNs were able to gradually release zinc ions to the medium and also showed an enhanced ability to adsorb proteins.^10^ Among the different bioactive metal ions tested, strontium has been extensively investigated in the context of bone repair materials due to its structural and physicochemical similarity to calcium, promoting bone regeneration and inhibiting bone resorption.^10^, ^36^, ^37^, ^38^ Lei et al. developed a SrHAP/chitosan (CS) nanohybrid scaffold by freeze‐drying technology. The SrHAP nanocrystals can uniformly disperse into the scaffolds and, with the release of Sr^2+^ ions, cell proliferation, and osteogenic differentiation can be improved. Additionally, the presence of strontium in the scaffolds promoted ECM mineralization, alkaline phosphatase (ALP) activity, and expression of the osteogenic genes ALP and COL‐1.^39^

Since the 1970s, the possibility of using silicon, usually acting as the silicate ion (Si^4−^), for bone formation has emerged.^40^ Si has a critical role in the metabolism of bone formation and is utilized to induce hydroxyapatite precipitation into the matrix by elevating its concentration at the early stages of bone calcification.^33^ Mao et al. *c*reated bioactive bone regeneration particles (BRPs) using amorphous calcium phosphate and 58S bioglass, composed of β‐tricalcium phosphate (β‐TCP) and calcium silicate, that could enhance bone regeneration. The BRPs also showed outstanding osteoinduction and osteoconduction for alveolar bone repair.^41^

Magnesium accounts for approximately half the mineral complement of bone tissue.^42^ It is also essential for many metabolic reactions.^43^ Among many effects of magnesium ions, its direct influence on osteogenesis is significant. Yoshiwaza et al. found that Mg^2+^ improved ECM mineralization in human bone marrow stromal cells (BMSCs), enhancing the expression of collagen‐X and vascular endothelial growth factor (VEGF).^44^ Hung et al. demonstrated that Mg^2+^ initially induces an osteogenic effect in the marrow cavity before motivating BMSC differentiation into osteoblasts through activation of the canonical Wnt signaling pathway. They further demonstrated the effective application of Mg‐based devices in therapy, especially in the bone regeneration field.^45^ Minardi et al. *c*onstructed a bio‐inspired biomimetic osteogenic niche with osteoinductive potential, composed of magnesium‐doped hydroxyapatite/type‐I collagen, which represents a critical advance in acellular off‐the‐shelf substitutes for bone regeneration applications.^46^

Calcium is the most abundant mineral in the body and is stored mainly in the skeleton.^47^ During the process of bone remodeling, the extracellular calcium ion concentration can be elevated to some extent through bone‐resorbing osteoclasts.^48^ This resorption can be inhibited, as can be the proliferation and differentiation of MSCs^49^, ^50^, ^51^ and the osteoblasts can be stimulated.^52^, ^53^ During the 1980s, researchers found that an extracellular G‐protein‐coupled receptor, namely the calcium sensor receptor (CaSR),^54^ can be activated, resulting in increased levels of calcium, which are then able to promote proliferation, chemotaxis, and osteogenic differentiation of BMMSCs in a dose‐dependent manner.^55^ In view of the composition of natural bone and the significant role of calcium in cellular activities, diverse materials composed of calcium phosphate have emerged as bone substitute treatments.^56^, ^57^, ^58^ However, although the deposition of calcium phosphate on the surfaces of these bone replacement materials may benefit osseointegration, the calcium deficiency remains a problem in bone regeneration.^59^ It is known that ionic dissolution products have beneficial effects on cellular activities, suggesting that dissociated calcium and phosphate ions may promote the osteogenic differentiation of osteoblasts.^60^

Because of the beneficial antimicrobial properties of the silver ion (Ag^+^) in tissue regeneration applications, incorporation of Ag^+^ into tissue engineering scaffolds could be useful to inhibit infections with minimal adverse effects.^32^ Qing T. et al. found that silver‐based nanoparticles were able to facilitate the proliferation and differentiation of MC3T3‐E1 cells, further contributing to the upregulation of bone formation and regulation markers.^61^ 3D scaffolds incorporating AgNP‐loaded nHA@RGO have been investigated. These composite scaffolds have been shown to effectively eliminate infection and inhibit the formation of biofilm, further facilitating bone repair.^62^

Iron is indispensable for a wide variety of cellular processes in the human organism,^63^, ^64^, ^65^ including the synthesis of DNA, RNA, and proteins, as well as electron transport processes, cellular proliferation, and differentiation.^66^, ^67^ In bone regeneration, in vitro experiments have shown inhibition of osteogenic lineage differentiation in human osteoblasts concomitant with decreased calcification caused by iron overload.^68^, ^69^, ^70^ Furthermore, in vivo studies in zebrafish larvae have shown reduced osteoblast function and mineralization caused by iron overload resulting in the augmented generation of reactive oxygen species.^59^ Deferoxamine, an iron chelator able to remove iron throughout the body, is able to counteract this effect in osteoblasts progenitors and has been applied extensively in osteogenesis.^70^ However, in vivo experiments found that the exposure of BMSCs to high iron concentrations may have negative effects such as impairing differentiation toward the osteogenic lineage. In contrast, Wang et al. reported positive effects of iron oxide nanoparticles(IONPs), mediated by MAPK signaling on the osteogenic differentiation of human BMSCs in vitro.^71^ Furthermore, Zhao et al. demonstrated the impacts of both low and high iron concentrations on osteoblasts.^72^ They found that osteoblastic differentiation was inhibited as the increase of iron concentration in a concentration‐dependent manner while a mild iron deficiency caused an elevation in cellular activity. However, osteoblastic differentiation may be restricted at critically low iron levels. Consequently, the potential advantages of iron in tissue regeneration need further exploration.

## 3 THE POTENTIAL OF DECELLULARIZED EXTRACELLULAR MATRIX SCAFFOLDS

The extracellular matrix (ECM) is a complex network of structural and functional molecules secreted by cells.^73^ All tissues and organs are thus largely composed of cells and ECMs. The main components of the ECM are (i) proteoglycans and glycosaminoglycans (GAGs), (ii) filamentous proteins such as collagen and elastin, (iii) adhesive proteins such as laminin, vitronectin, and fibronectin. Bone ECM has both inorganic and organic constituents. The inorganic part, consisting of calcium phosphate, mainly in the form of hydroxyapatite (HA), is the source of bone strength,^74^ while the organic part, composed mostly of type I collagen, provides the tissue and cell with flexibility and adhesion, respectively. Decellularized bone is frequently used as a special scaffold material in bone tissue engineering, due to its ability to eliminate cellular components and antigenicity and its osteogenic and biomechanical properties as well as its physiological similarity to the bone matrix.

**FIGURE 3:**
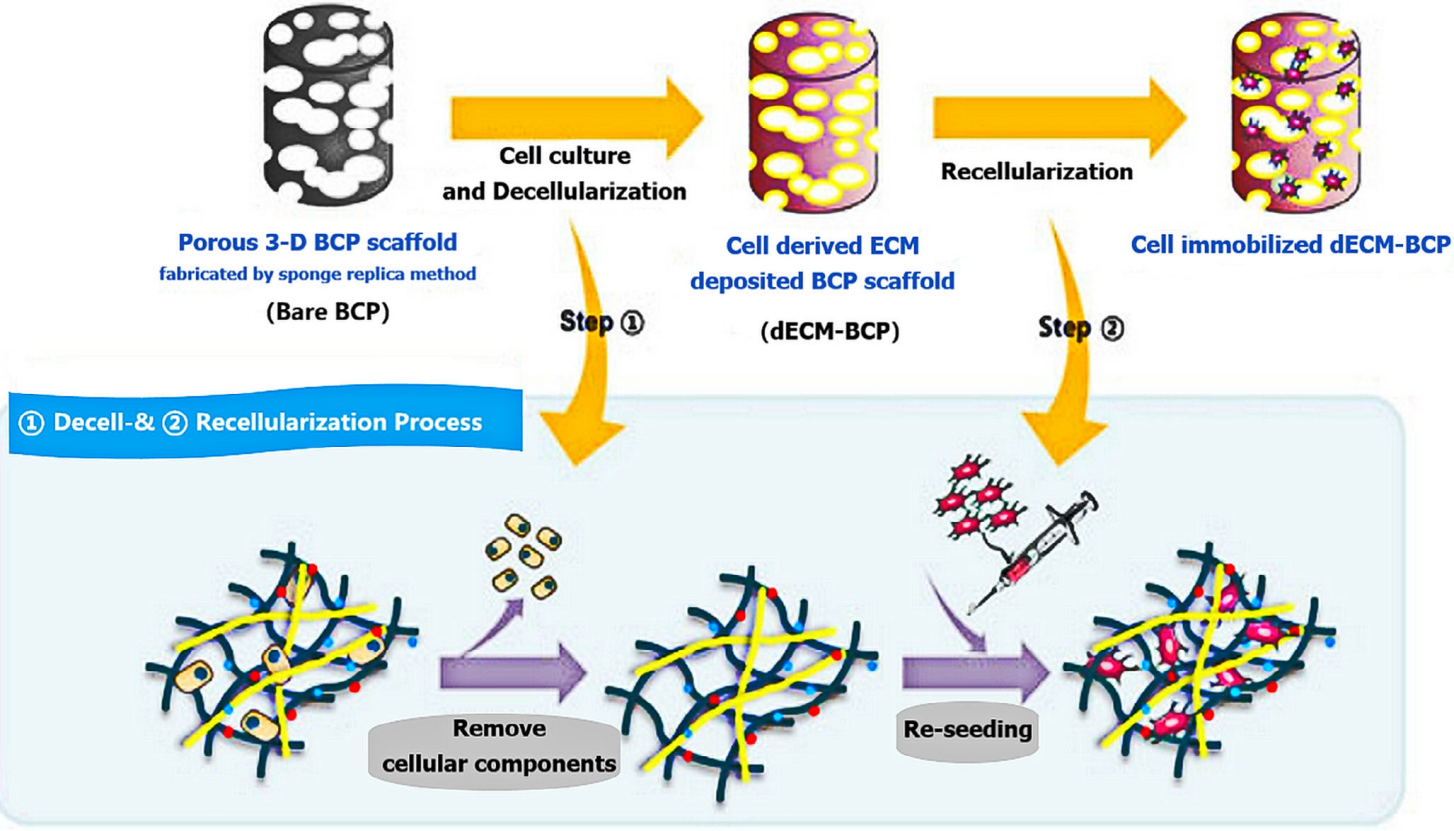
Schematic diagram of the procedure to generate cell‐derived extracellular matrix deposited on biphasic calcium phosphate (BCP) scaffolds. The rat‐derived bone marrow mesenchymal stem cells were seeded on BCP scaffolds, cultured for 1 week in growth medium, and incubated for 3 weeks with osteogenic medium followed by decellularization using freezing and thawing and sodium dodecyl sulfate treatments. The decellularized extracellular matrix‐biphasic calcium phosphate (dECM‐BCP) scaffolds were evaluated in vitro

Boram et al. demonstrated that the potential of a biphasic calcium phosphate (BCP) scaffold with attached dECM in bone tissue engineering. Rat BMSCs were cultured on porous BCP scaffolds for 3 weeks, after which the decellularized ECM‐deposited scaffolds (dECM‐BCP) were further utilized for study in vitro (Figure 3). The results indicated that the BCP scaffold with ECM was enhanced the bioactivity of the materials, as well as offering a stable microenvironment for osteogenesis.^13^ Wang et al. showed that adipose‐derived ECM (A‐ECM) could be combined with chitosan/gelatin conducive to the attachment and growth of BMSCs. Thus, for ECM scaffolds with poor mechanical properties, the association of chitosan/gelatin with the ECM can promote not only the strength of the ECM scaffolds but also the bioactivity of composite scaffolds, while simultaneously enhancing the osteogenic ability of chitosan.^12^

In contrast to decellularized ECM scaffolds, Platelet‐rich fibrin (PRF), which functions as a growth factor vector, has been widely used in the field of soft and hard tissue regeneration.^75^, ^76^, ^77^ However, the bioactive stability of decellularized PRF (DPRF) is unknown. Chi et al. investigated whether the incorporation of DPRF into the chitosan/gelatin scaffold could synergistically improve both the bioactivity of the C/G scaffold and the strength of PRF, due to the suitable biocompatible and mechanical properties of C/G scaffolds, but found a lack of bioactivity. Ultimately, the merging of DPRF can not only promote BMSC adhesion, proliferation, and osteogenic differentiation with a suitable microenvironment in vitro but also expedite bone repair in vivo.^78^

## 4 MICRO/NANO‐STRUCTURAL FEATURES OF THE BIOMIMETIC SCAFFOLD

Tissue engineers need to mimic the micro/nano‐architecture of natural bone to investigate the means of stimulating effective tissue growth. To understand the intrinsic osteoinduction of materials, probing the tunable structural properties of scaffolds is necessary. Of these features, primary concern are gross features such as the surface roughness and morphologies of materials on which cells proliferate and attach, in addition to substrate modulus and pore size conducive to osteogenic differentiation, and distinctive structural components such as fibrils of particular sizes and interconnectivity.^79^

### 4.1 Nanostructured surfaces and interfaces

**FIGURE 4:**
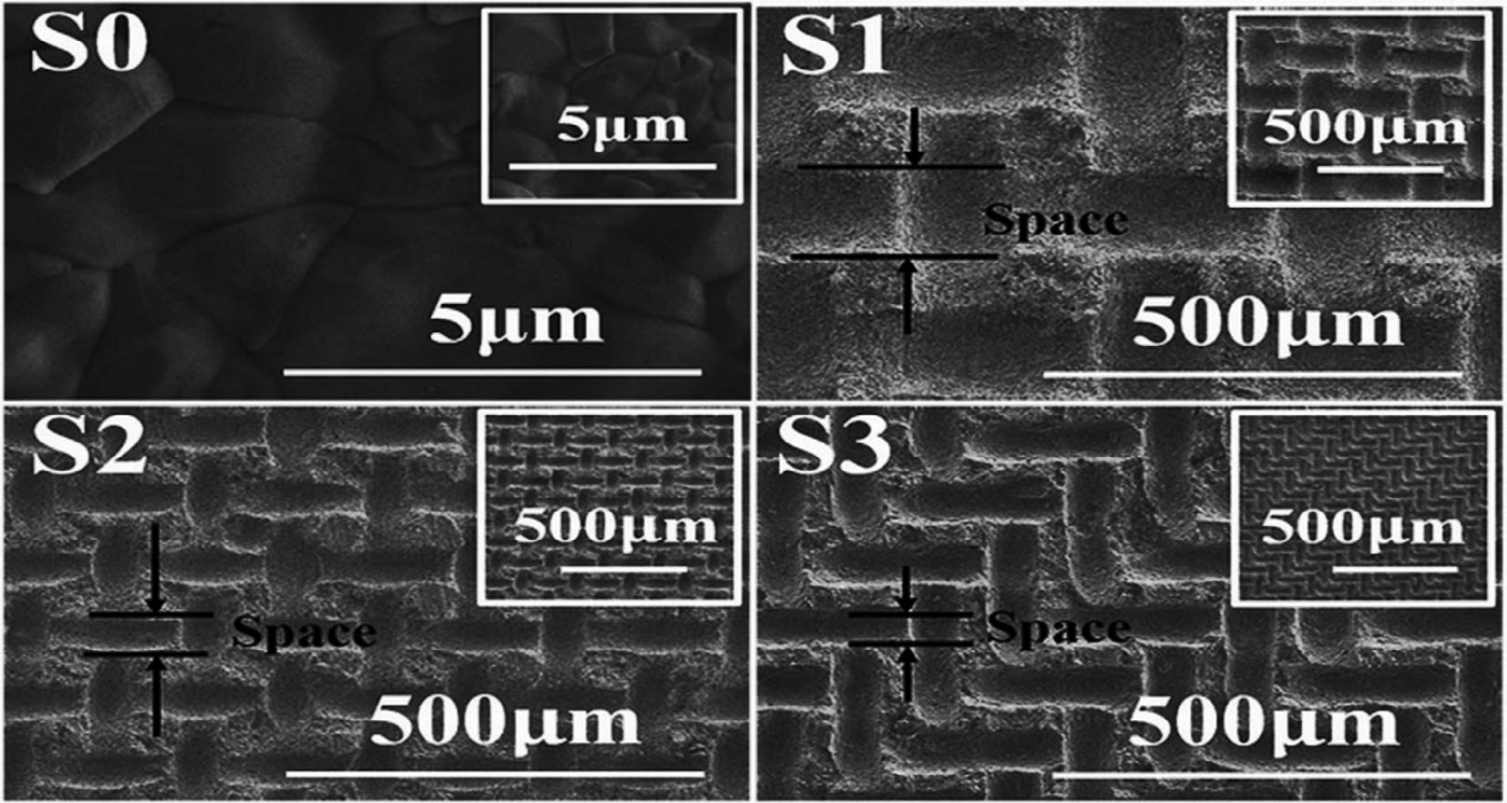
FESEM images of the HAp bioceramic surfaces with a flat surface (S0) as the control sample, and the micropatterned surfaces using 100 (S1), 200 (S2), and 400 (S3) mesh nylon sieves as templates. The inserted small figures in the top right corner are low magnification images, and the mark of the “space” denotes the distance between the adjacent convexes *Source*: Adapted with permission (Qiu et al.^87^)

The design of materials to direct cell behaviors and thus to promote tissue repair is of great concern in tissue engineering and is essential for the improvement of bioactive materials.^80^, ^81^, ^82^ MSC differentiation may be triggered by surface topography or by the release of growth factors, calcium, and phosphate by inflammatory cells such as macrophages, monocytes, and osteoclasts.^83^ On the microscale, the ECM of bone is in the form of planar lamellae composed of collagen fibers with HA deposits. Previous studies have shown that rough surfaces with micro−/nanostructured topographies and patterned surfaces can significantly enhance the biological efficacy of the materials for cells.^84^, ^85^, ^86^, ^87^ Zhao et al. created convex micropatterns of different sizes on a hydroxyapatite bioceramic surface, using a patterned nylon sieve as the template (Figure 4). These surfaces provided superior wettability and surface energy with significantly improved effects on rat bone marrow stromal cell (bMSC) proliferation, adhesion, and osteogenic differentiation. The author further showed a much better stimulation of the cell response with surface pattern sizes that were similar to the cell size, compared with larger pattern sizes.^88^ Xia et al. designed Si‐substituted HAp bioceramic scaffolds with specific nanosheet and nanorod structures using hydrothermal treatment of calcium silicate. It was found that these surfaces were able to promote cell attachment and spreading as well as stimulating proliferation and osteogenic differentiation in rBMSCs. These effects were enhanced by the incorporation of Si, with the best effects produced using a Si‐substituted HAp bioceramic with a nanorod surface.^89^

### 4.2 Micro/nano‐porous structures of the scaffolds

**FIGURE 5:**
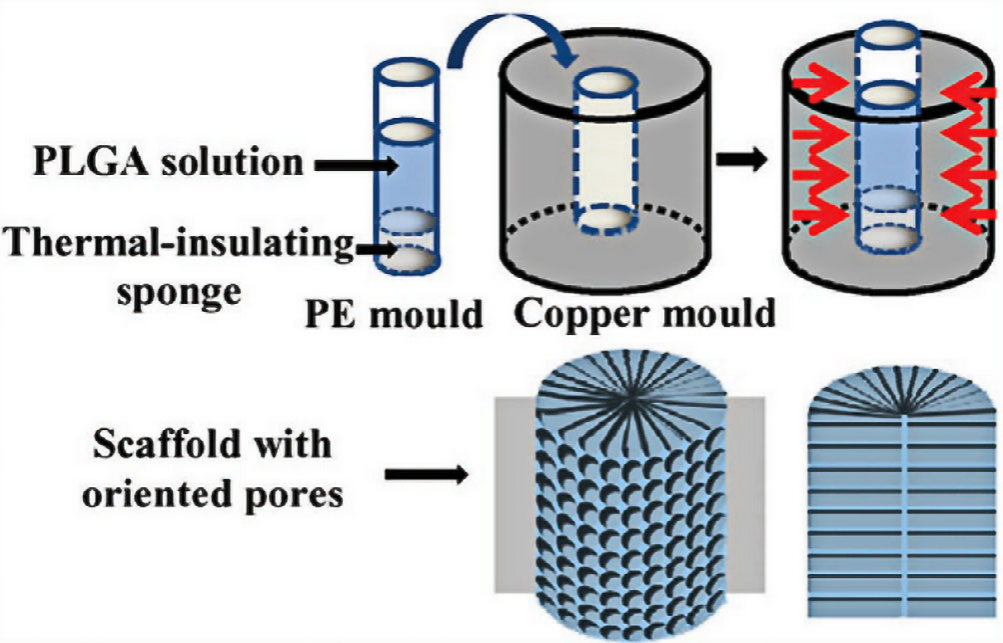
Schematic illustration to show the preparation procedures of an oriented porous O‐HA‐MA/PLGA scaffold by cooling in the radial direction. The HA‐MA solution was placed in a PE mold with the bottom attached to a thermal‐insulating sponge to avoid heat exchange from below. The PE mold was placed in a precooled copper mold to allow crystallization of the solvent from the radial direction. The cartoons on the lower right side show the 3D and side view of the structured scaffold *Source*: Reprinted with permission (Zhang et al.^95^)

Bone tissue engineering scaffolds require interconnected 3D pore structures that permit cell infiltration as well as allowing nutrient access and waste removal.^90^ Most studies describe surfaces that only permit one‐way guidance, resulting in only transverse or longitudinal migration of cells and asymmetric repair of the tissue defect.^91^, ^92^ Thus, a necessity for symmetrical regeneration is facilitated migration of the cells into the center of scaffolds. It has been found that scaffolds with oriented porous structures are effective in this regard^93^, ^94^ as oriented pores are able to promote cell infiltration allowing improved tissue regeneration both in vivo and in vitro.^95^ Dai et al. reported an O‐HA‐MA/PLGA scaffold with radial pores prepared by directed cooling, freeze‐drying, and PLGA infiltration (Figure 5). It was found that this type of scaffold allowed bone marrow stem cell (BMSC) aggregation characterized by spherical cell morphology, while the cell‐free hybrid scaffold facilitated regeneration by the recruitment of surrounding BMSCs and chondrocytes rather than preseeding any type of cells.^96^ Shin et al. demonstrated the efficacy of radially aligned fibrous scaffolds (RAFSs) with PLLA‐coated polydopamine in promoting the directional migration of human mesenchymal stem cells (hMSCs). The RAFSs were composed of fibers distributed radially from the periphery to the center, with the polydopamine coating enhancing cell migration. The radial fiber distribution of the scaffold provided direction to the hMSC migration while modulating the cell shape to become elongated and oriented toward the scaffold center.^97^

### 4.3 3D scaffold materials with irregular and hierarchical structures

Treatment of complicated and irregularly shaped bone defects and the complications of various bone diseases remains a clinical challenge.^98^ Although there are a variety of products, including bone void filters and temporary scaffolds as well as reports of new biomaterials, many of these have surgical, technical, and manufacturing shortcomings.^99^, ^100^ Evidence indicates that inadequate contact with host bone tissue adversely affects osseointegration.^101^ Ceramics and cements, both natural and synthetic, are frequently used for the clinical treatment of bone defects;^99^ however, rigid ceramics are difficult to machine, indicating that ceramic constructs cannot be simply shaped and tuned in clinical treatment to accommodate the defect site.^102^ In addition, the fragile and brittle nature of ceramics has restricted their application. Recently, a novel material allowing shape recovery has appeared to be a groundbreaking application in regeneration medicine.^103^ The shape recovery feature guarantees scaffold implantation in a compressed form with minimally invasive surgery, avoiding the technical, surgical, and manufacturing limitations and allowing the scaffold to fit into the defect site. Moreover, as native bone ECM has a nanofibrous physical structure with 65–70 wt% inorganic composition,^104^, ^105^, ^106^ the 3D scaffolds resemble native physicochemical structures, which are composed of inorganic nanofibrous with shape‐recovery features, and thus could have potential for bone tissue regeneration.

**FIGURE 6:**
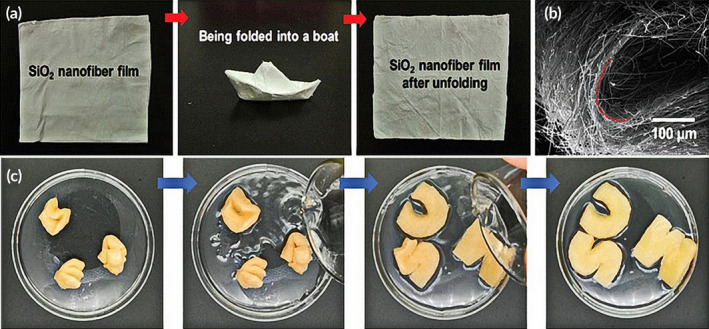
3D nanofibrous scaffolds from flexible inorganic nanofibers. (a) Flexible SiO_2_ nanofibers with foldability. (b) SEM image of a curved SiO_2_ nanofibrous mat showing that SiO_2_ nanofibers can achieve 180° deflection without fracture. (c) The shape recovery process of SiO_2_ NF–CS scaffolds induced by hydration *Source*: Adapted with permission (Correia and Mano^109^)

**FIGURE 7:**
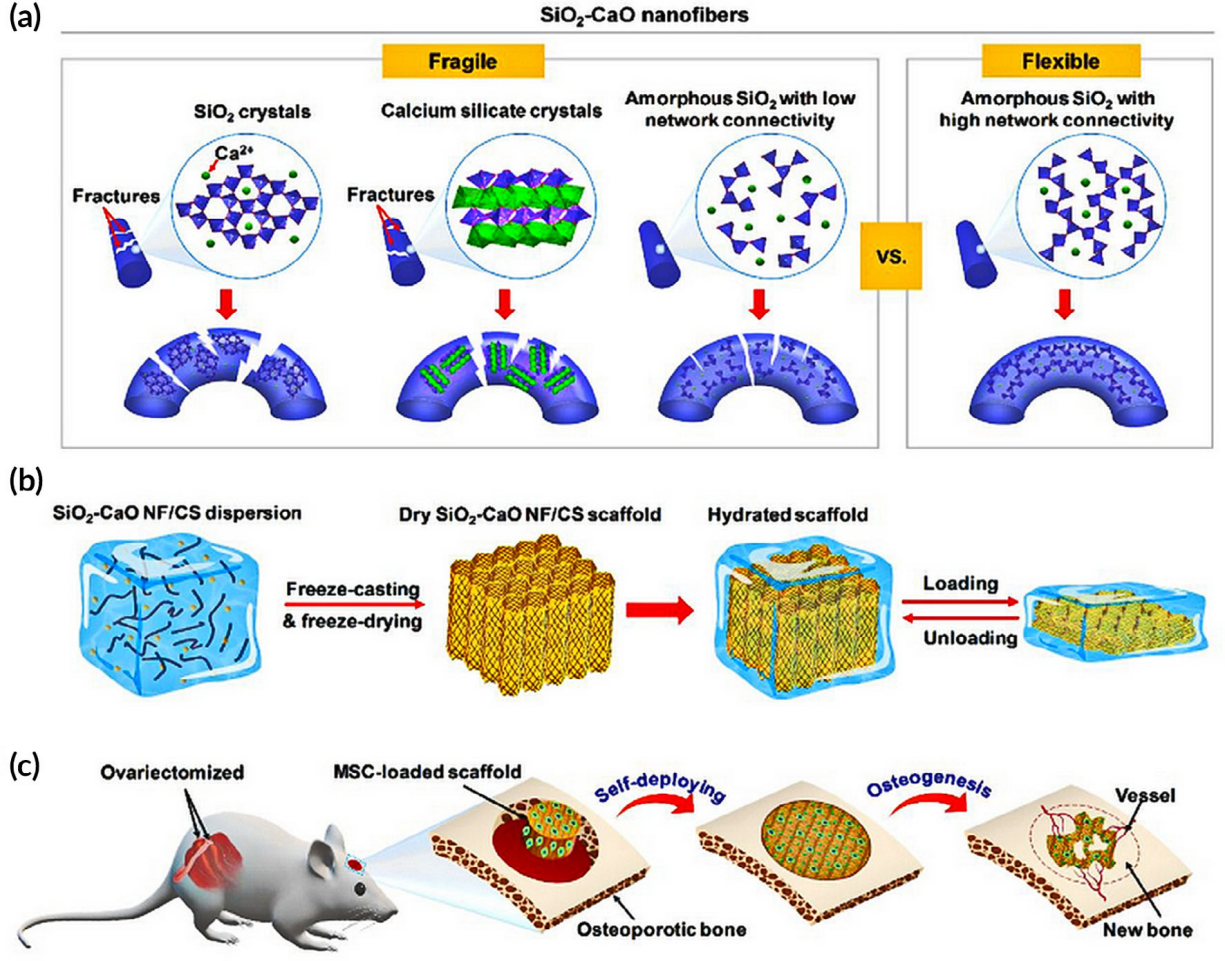
Schematic diagram of the production of an elastic 3D SiO_2_–CaO NF/CS scaffold to improve bone repair in osteoporotic rats. (a) Development of SiO_2_–CaO nanofibers with superior flexibility. (b) Generation of 3D SiO_2_–CaO NF/CS scaffold with excellent elasticity. (c) Repair of cranial defect in an osteoporotic rat via minimally invasive transplantation of the self‐deploying scaffold *Source*: Reprinted with permission from L. Wang, Qiu, Y. Guo, et al. Smart, elastic, and nanofiber‐based 3D scaffolds with self‐deploying capability for osteoporotic bone regeneration. *NanoLett*. 19 (2019) 9112–9120.^110^ Copyright (2019) American Chemical Society

Wang et al. prepared 3D superelastic scaffolds composed of flexible inorganic nanofibers that are capable of self‐fitting. First, flexible SiO_2_ nanofibers were encapsulated with chitosan (CS) layers using SiO_2_ NF–CS bonding points. Chitosan has the advantages of being biocompatible, biodegradable, and antibacterial and is thus widely utilized for tissue engineering.^107^, ^108^ Furthermore, chitosan undergoes a glass transition on hydration, endowing the chitosan scaffolds with shape recovery properties.^109^ The prepared SiO_2_ NF–CS scaffolds show shape recovery on hydration as well as good elasticity and resistance to fatigue. They have been shown to be effective in promoting hMSC differentiation in vitro as well as self‐fitting to bone defect sites and promoting bone regeneration in vivo^110^ (Figure 6). Second, SiO_2_–CaO glass nanofibers have excellent flexibility and bioactivity through their ability to modulate crystallization and chain configuration and this overcoming their inherent fragility (Figure 7a). Furthermore, the elastic SiO_2_–CaO nanofibers are divided and assembled into 3D porous scaffolds wrapped in the natural polymer chitosan using homogenization and lyophilization (Figure 7b). The SiO_2_–CaO nanofiber/chitosan (SiO_2_–CaO NF/CS) scaffolds are elastic allowing for shape recovery and biomineralization. These properties result in enhanced regenerative capability as demonstrated in vivo (Figure 7c)，where the SiO_2_–CaO NF–CS scaffolds enhanced both bone regeneration and revascularization.^111^ The above‐mentioned two strategies for fabricating elastic and shape‐recovery porous 3D scaffolds utilizing flexible inorganic nanofibers with self‐fitting ability and allowing minimally invasive surgery are promising strategies for innovative bone regeneration scaffolds.

## 5 PHYSICAL FACTORS AND INDUCEMENT‐REACTIVE SCAFFOLDS

**FIGURE 8:**
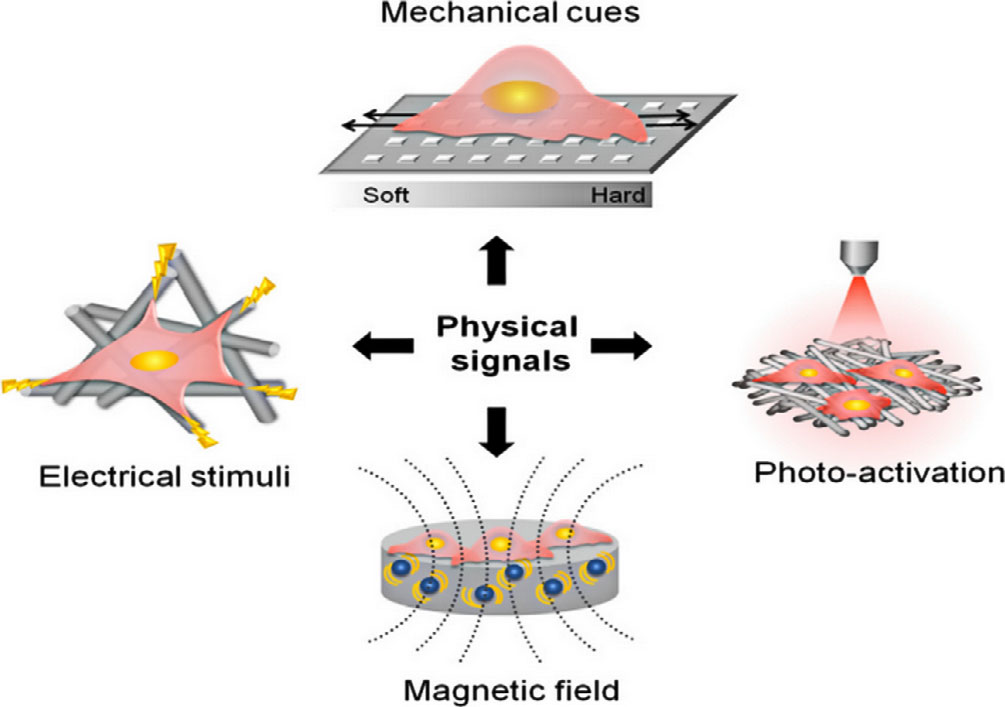
Illustration of the physical signals (mechanical, electrical, magnetic, and photic stimuli) and intrinsic cues of scaffolds synergistically affecting cellular behavior *Source*: Adapted with permission (Du et al.^31^)

The strategies of constructing acellular scaffolds involve not only the optimization of internal structures, surface modification, and growth factor delivery of the 3D biomaterials, but also a consideration of external physical cues, including electrical, mechanical, and magnetic stimuli, as well as photothermal drive can influence biological processes, including bone regeneration.^21^, ^112^, ^113^ Thus, synergistic regulation of bone repair has been employed, combining external physical stimuli with the internal structures of scaffolds, in particular, those features that are responsive to stimuli (Figure 8).

### 5.1 Photothermally‐controlled bone scaffolds

Recently, photothermal therapy (PTT) has been utilized for eliminating tumors and stimulating tissue regeneration.^114^, ^115^, ^116^, ^117^, ^118^ PTT and radiotherapy (RT) are extensively employed in clinical treatment where, in combination with nanomedicine, they have proved successful.^119^, ^120^ Various biomaterials with photothermal effects have been reported past a few years involving gold nanoparticles^121^, ^122^, ^123^ and carbon nanomaterials.^124^, ^125^, ^126^ Among these, graphene oxide (GO) has shown great promise due to its ability to absorb near‐infrared (NIR) light, its high photothermal transforming efficiency, and biocompatibility.^127^, ^128^, ^129^, ^130^ Yanagi et al. demonstrated that a carbon nanotube under a photothermal effect activated by NIR light promoted osteogenic gene expression in preosteoblasts. in vivo results indicated that the bone regeneration of calvarial defects was enhanced in comparison to controls not treated with NIR.^131^

**FIGURE 9:**
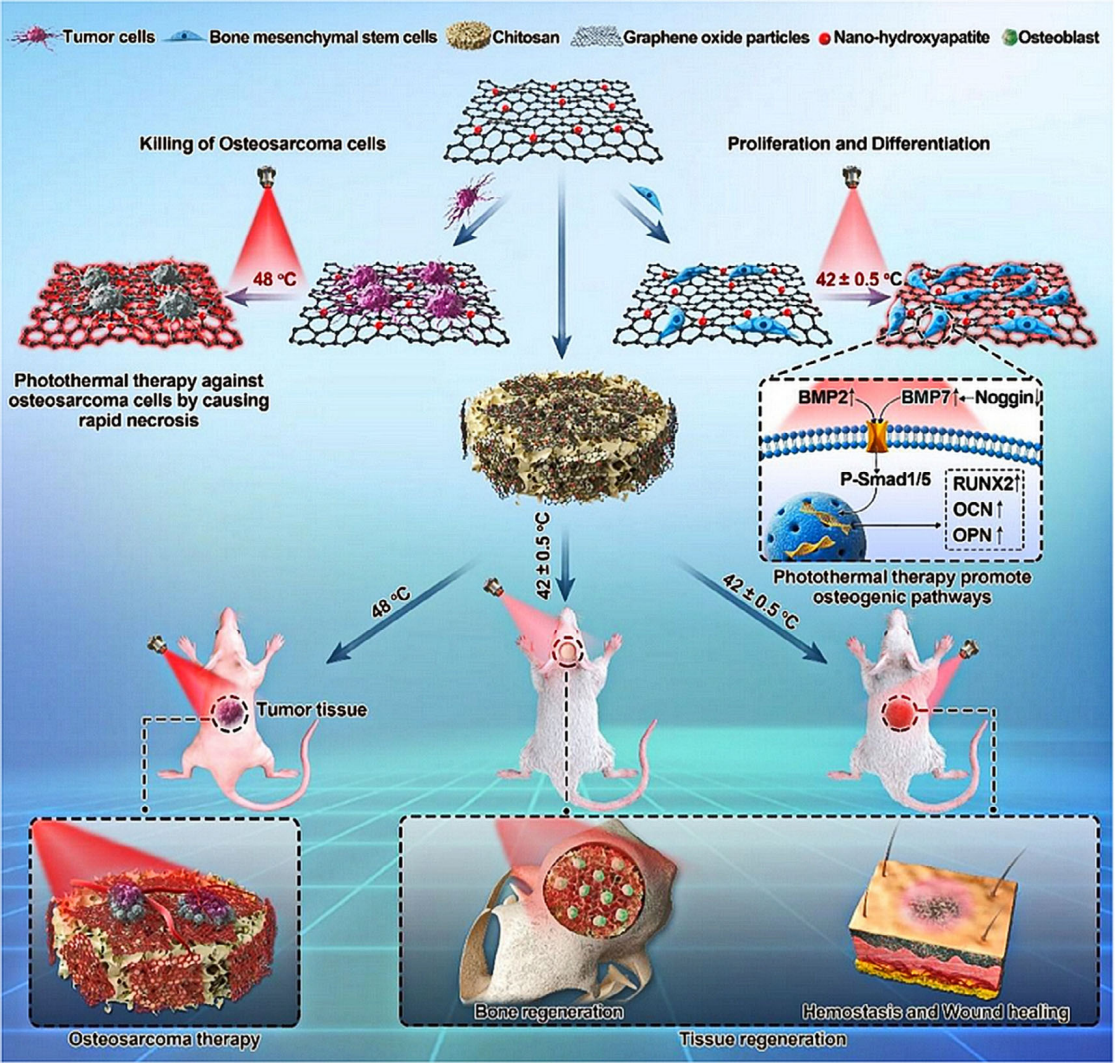
Schematic illustration of the construction of nHA/GO particles, nHA/GO/CS scaffolds, and their bio‐applications *Source*: Adapted with permission (Ma et al.^30^)

Photothermally controlled scaffolds have progressed toward multifunctionality with the capacity of addressing both challenges involving bone regeneration and the elimination of osteosarcoma cells. Ma et al. designed a bifunctional CS scaffold coated with nHA and GO, investigating its ability to eliminate osteosarcoma cells and improve MC3T3‐E1 cells, as well as the effects of NIR irradiation on hBMSC proliferation and differentiation. Furthermore, the underlying mechanism of osteogenesis was discussed (Figure 9). It was found that appropriate proportions of nHA and GO produced better effects. In this study, 30% of nHA and GO improved the biocompatibility, while also showing an excellent photothermal effect in removing HOS and promoting differentiation of hBMSCs. For the evaluation of in vivo tissue regeneration, results from the micro‐CT images and tissue staining indicated that nHA/GO/CS showed significantly higher new bone formation compared to controls.^30^ In a similar report, a bioceramic scaffold was modified through bioprinting with self‐assembled Ca‐P/polydopamine nanolayers. With the photothermal effect of polydopamine, the scaffolds significantly induced apoptosis of tumor cells in vitro and inhibited tumor progression in vivo. Due to the nanostructural surface, the scaffold was shown to enhance new bone formation with photothermal stimuli in a rabbit model.^132^

With the exception of the bifunctional scaffolds with the photothermal effect, the development of thermodynamically controllable bone‐like hierarchically staggered architecture has been reported, including the construction of a high‐energy polyacrylic acid‐calcium intermediate, which drives mineralization in collagenous‐deficient gap areas by an energetically downhill process. Liu et al. aimed to demonstrate the mechanism of formation of the staggered architecture from a thermodynamic viewpoint and produced osteoinductive materials similar to autografts with equivalent effects on cell homing, osteogenic differentiation, and treatment of bone defects. The selective mineralization process in the collagenous gap areas was modulated by a high energy level of PAA‐Ca media through an energetically downhill process. Among the three groups, it was found that HIMC, with structural and functional features similar to native bone tissue, provided a beneficial microenvironment for cell homing and multidifferentiation, while recruiting native stem cells for bone defect repair.^29^

### 5.2 Electrically and magnetically guided bone scaffolds

Since the discovery of the bioelectrical properties of bone in the 1950s, electrical stimuli have been recognized in clinical therapy as a supplement to speed fracture healing and promote spinal fusion.^20^, ^133^, ^134^ Because of the presence of electric current and potential in native bone and periosteum, it has been suggested that they play an essential role in sustaining bone quality and volume.^135^, ^136^ Several capacitive biomaterials capable of storing electrical charge on their surfaces have been discovered. These biomaterials have shown promise in the bone regeneration field. Recent findings have demonstrated that electrical stimuli can drive bone cells to migrate, proliferate, and differentiate at particular sites in vitro.^137^, ^138^, ^139^, ^140^ Clinical results also indicated that electrical stimuli could boost bone healing via interactions between bioelectrics and charged biomolecules.^135^, ^140^ Bandyopadhyay et al. explored the innovatory combination of bioactive TiO_2_ nanotubes (TNT) with charge storage to modify the surface of Ti (CpTi) on enhancing bone cell‐material interactions in vitro and osseointegration in a rat distal femur defect model in vivo. The results indicated that the polarized TiO_2_ TNTs grown on the Ti surface promoted osteoblast adhesion, proliferation, and differentiation, showing the effect and biocompatibility of TNT‐P toward optimizing interactions between surrounding cells and materials in vitro. Histological and SEM results showed accelerated healing and interfacial bonding between the implant and the bone tissue.^141^

**FIGURE 10:**
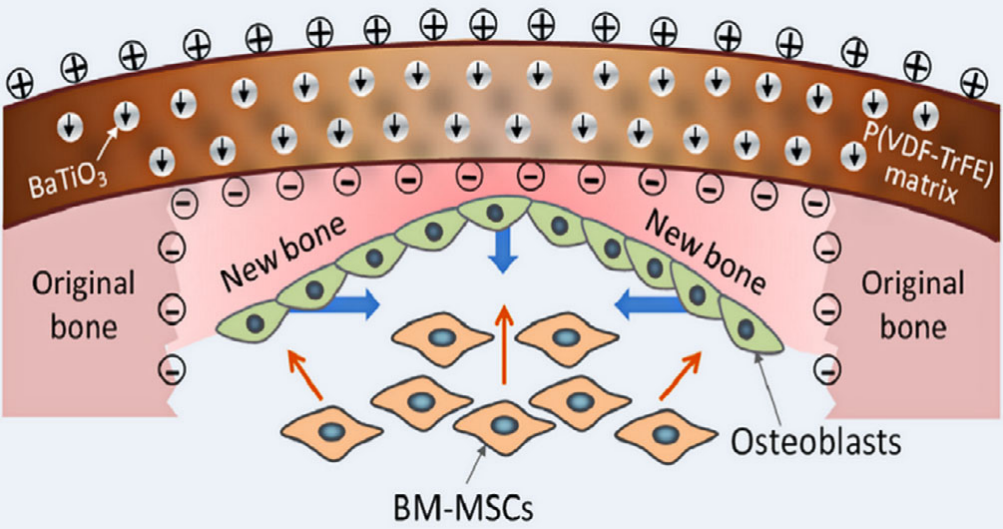
Illustration of the biomimetic electric microenvironment created by BTO NP/P(VDF‐TrFE) composite membranes promoting bone defect repair *Source*: Reprinted with permission from X. Zhang, C. Zhang, Y. Lin, et al. Nanocomposite membranes enhance bone regeneration through restoring physiological electric microenvironment. *ACS Nano*. 10 (2016) 7279–7286.^23^ Copyright (2016) American Chemical Society

Since electric microenvironment‐stimulated wound repair has been proposed as a cue for bone regeneration, recovery of a damaged physiological potential microenvironment appears to be an effective strategy for bone regeneration.^142^ Zhang et al. designed biomimetic electric microenvironment nanocomposite membranes with homogeneous scatter of ferroelectric BaTiO_3_ nanoparticles (BTO NPs) in polyvinylidene fluoridetrifluoroethylene (PVDF‐TrFE) matrix (Figure 10). As the nanocomposite membranes cover the bone defect site like native periosteum, surrounding BMSCs can be attracted by galvanotaxis and stimulated to differentiate into osteoblasts through the bioelectric potential produced by the composite membranes. Furthermore, the surface potential of the membranes was found to be stably conserved having more than half of its original surface potential 12 weeks after implantation.^23^

**FIGURE 11:**
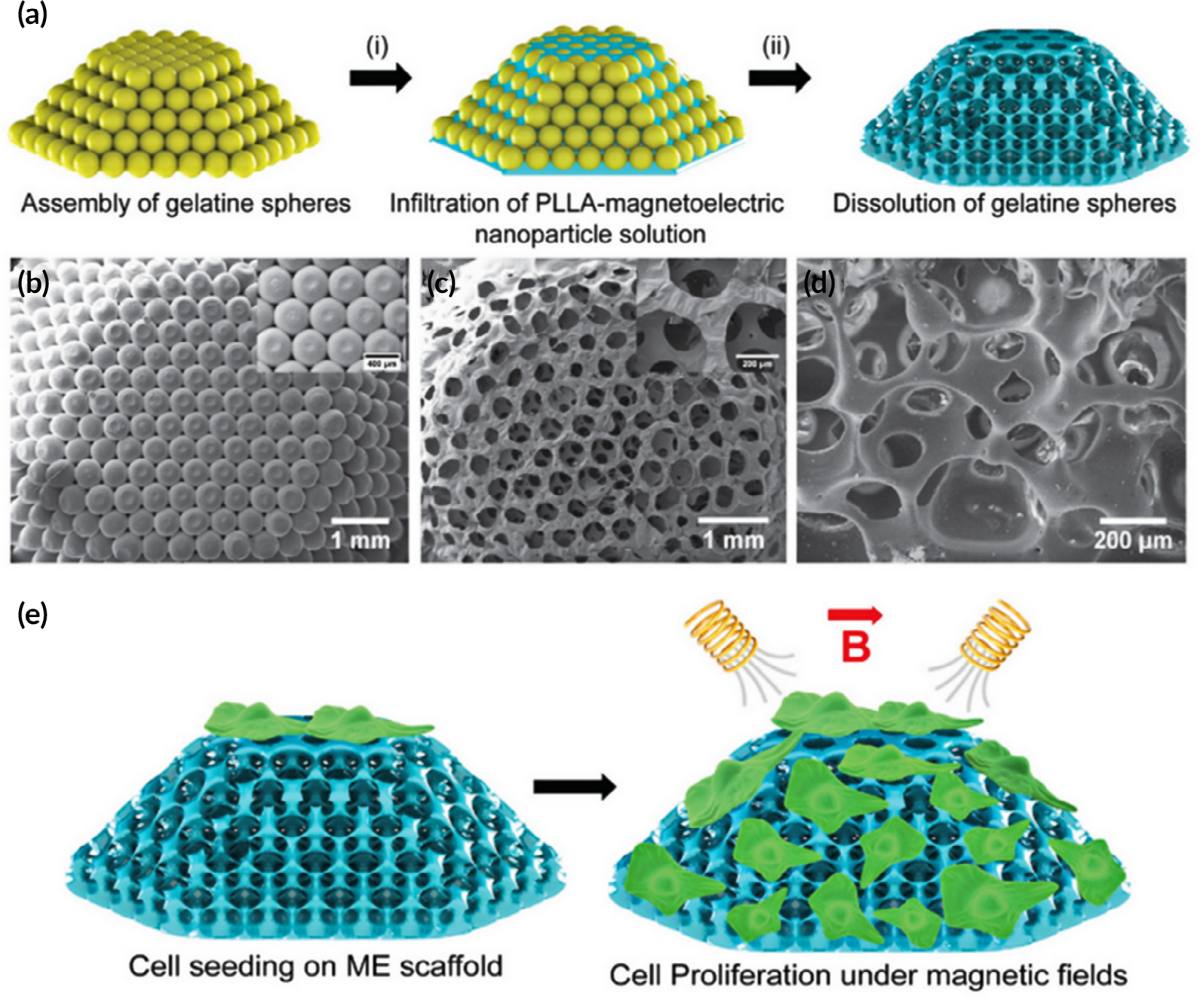
Construction of magnetoelectric (ME) inverse opal scaffolds and structural characterization of ME NPs and the ME scaffold. (a) Scheme showing the construction steps starting with the assembly of gelatin spheres, followed by (i) their infiltration with a solution of PLLA and ME nanoparticles and (ii) the removal of gelatin spheres to obtain 3D and porous ME scaffolds. SEM images of (b) assembled gelatin template and the inset show its magnified image. (c) SEM image showing the top view of a 3D ME scaffold and the inset shows its magnified image demonstrating a uniform porous structure. (d) SEM image presenting a cross‐sectional view of a uniform and well‐connected ME scaffold. (e) Scheme showing the ME effect induced enhanced cell proliferation on 3D scaffolds under the influence of AC magnetic fields *Source*: Adapted with permission (Mushtaq et al.^19^)

Various piezoelectric materials have been used for creating electroactive scaffolds that have capacities of wirelessly electrostimulating cells under mechanical stress,^143^, ^144^, ^145^ especially piezoelectric nanobiomaterials.^146^ Moreover, the utilization of wireless magnetic fields to activate piezoelectric scaffolds has received significant attention recently, owing to their minimally invasive character and the simplicity of manipulation. In this field, Mushtaq et al. designed 3D magnetoelectric inverse opal scaffolds composed of biodegradable PLLA and electromagnetic nanoparticles, imitating the naturally occurring porous and piezoelectric bone microenvironment by producing electric charges wirelessly. The effects of electric stimuli induced by magnetic field on the proliferation of MG63 osteoblast cells were determined using both two‐dimensional (2D) membranes and 3D scaffolds (Figure 11). Using the 2D membranes, a 40% increase in cell proliferation was observed compared to controls, while use of the 3D scaffolds resulted in a 134% increase in proliferation.^19^ Besides the application of electromagnetic effects for bone regeneration, pure magnetic stimulation treatment by pulse electromagnetic fields (EMF) has emerged in clinical therapy for bone healing for many years, though with limitations.^147^ in vitro studies have determined that static magnetic fields (SMF) and EMF could both promote osteoblast differentiation,^148^, ^149^ while in vivo findings also demonstrated that SMF and EMF can improve bone healing and enhance bone integration with grafts.^150^ It has been assumed that the underlying biological mechanism might be cell membrane deformation and cytoskeletal restructuring induced by magnetic stimuli, owing to the presence of water acting as a diamagnetic fluid, triggering mechanically stimulated signaling pathways to promote osteoblast differentiation.^151^ Magnetic tissue engineering scaffolds, especially magnetic bone scaffolds that have exhibited prospects for enhancing bone regeneration, have been constructed based on these natural biological reactions through embedding magnetic nanoparticles (MNPs) into diverse matrices.^152^, ^153^, ^154^ For instance, the embedding of MNPs into PCL nanofiber scaffolds indicated that the composite scaffolds not only enhanced the osteogenic differentiation of rat MSCs in vitro but also promoted vascularization and bone regeneration in vivo.^155^ However, the underlying mechanisms of interactive response between the magnetic scaffolds and cells or tissues remain obscure. One hypothesis is that the blended MNPs ameliorate physical properties, such as mechanical features, hydrophilicity, and degradation rate, thus enhancing cell adhesion and bone regeneration. Another possible reason may be the generation of an internal magnetic field induced by the incorporation of MNPs, thereby affecting cell behavior.

Besides the use of MNPs‐embedded magnetic scaffolds, the stimulation of external magnetic fields can be utilized to promote scaffold fixation^156^ and drive cells toward angiogenesis and osteogenesis.^157^ For instance, an external SMF applied on magnetic PCL/MNP scaffolds for osteoblast differentiation and bone formation has been studied showing that the external SMF stimuli not only facilitated osteoblast differentiation in vitro, but significantly promoted new bone regeneration in mouse skull defects, in comparison to magnetic scaffolds alone.^158^ In addition，an external magnetic field not only can stimulate cells toward bone regeneration but can produce a direct effect on angiogenesis. Sapir et al. demonstrated that an external alternating magnetic field prompted the formation of vessels in endothelial cells in magnetically reaction alginate scaffolds.^159^ However, the underlying mechanisms of how external magnetic fields motivate osteogenesis and angiogenesis are still unknown and it is assumed that microdeformation induced by magnetic scaffolds may produce bending and stretching forces that could mechanically stimulate cells.^157^, ^160^

### 5.3 Mechanically sensitive bone scaffolds

At the molecular level, cell behavior, for instance in MSCs, may well be regulated by the mechanical properties of materials as the capacity of a material substrate to either store or dispel cellular forces could contribute to a strong cue to cells interacting with it.^161^, ^162^, ^163^ Regarding bone which is composed of diverse cells and ECM and is a mechanically sensitive tissue, the effect of mechanical forces on the remodeling and structural improvement of bone has been long known.^164^ In particular, the effect of mechanical cues on cell behavior is important for sustaining bone tissue homeostasis.^151^ External mechanical forces have been suggested to be a potential of modulating MSC differentiation in vitro. As an example, a materials approach with a tunable rate of stress relaxation of hydrogels for the 3D culture of cells has been reported. The study aimed to investigate the effects of hydrogel substrate viscoelasticity and stress relaxation on cell proliferation, spread, and MSC differentiation under 3D conditions. It was found that with the initial elastic modulus of 17 KPa in fast‐relaxing hydrogels, MSCs could develop a mineralized, collagen‐1‐rich matrix resembling that of bone.^25^ Thus, the characteristic of cell mechanosensitivity to substrate through stress relaxation is a promising design parameter for biomaterials for bone repair. Another study demonstrated that mechanical stimuli in vitro could produce highly mineralized bone formation. Steinmetz et al. found that human MSC differentiation could be regulated by dynamic mechanical stimuli causing expression of collagen‐I as well as the formation of mineral deposits in the bone layer of an osteochondral hydrogel.^165^ Moreover, external mechanical stimulation can be combined with the natural physical characteristics of a scaffold for cell regulation. For instance, the patterns of aligned and unaligned nanofibers affecting MSC differentiation under tensile pressure have been used with good effect.^166^

**FIGURE 12:**
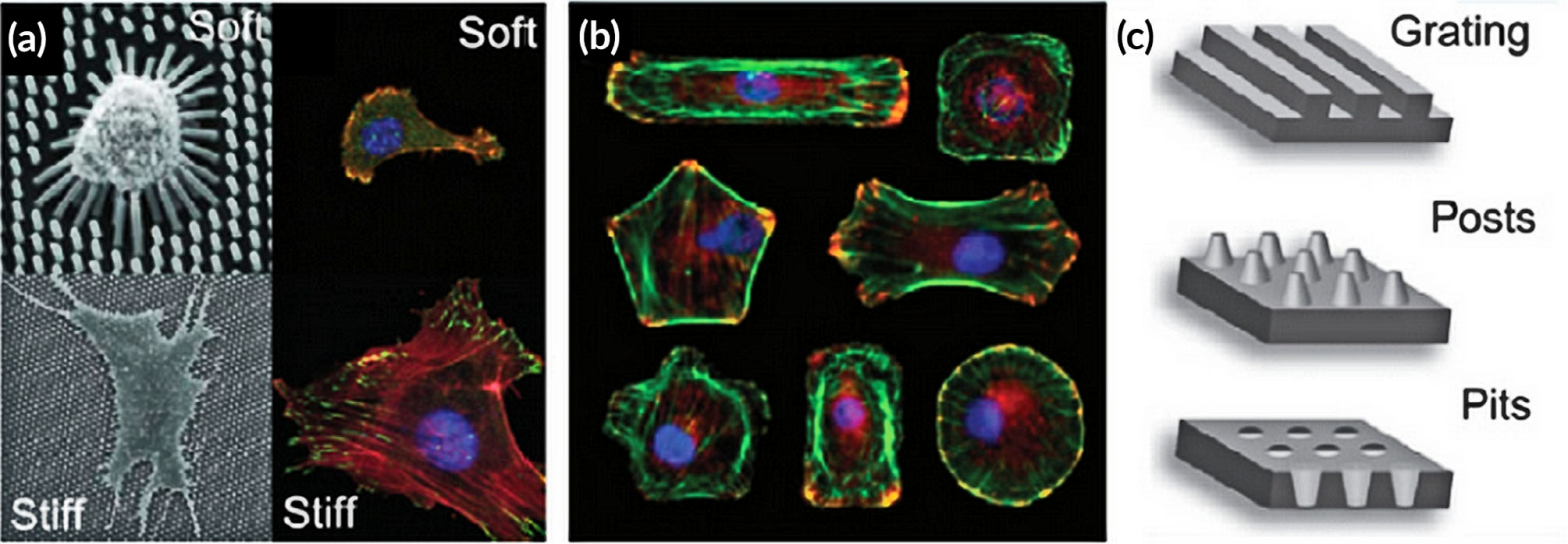
Mechanical cues created from substrates with different features, (a) stiffness, (b) micropatterning, and (c) nanotopography *Source*: Adapted with permission (Baker and Chen^167^)

Besides external mechanical factors, research has focused on the important role of intrinsic forces of the scaffold materials.^112^ Scaffolds that show beneficial mechanical signals via physical cues, such as stiffness and other mechanical properties, can generate internal mechanical forces to facilitate cell differentiation^167^ (Figure 12). For instance, on 2D membranes with 0.1–1 kPa stem cells developed into neurons. When grown on stiffer substrates (20–80 kPa), the cells had a greater probability of turning into bone cells.^168^, ^169^ It appears that the stiffness of the structure is more significant with a 3D substrate stiffness ranging from 0.7 to 3 MPa resembling that of cartilage being optimal for osteoblast functionality. In another study, Maggi et al. aimed to investigate the efficacy of stiffness of 3D nanoarchitected scaffolds on stress and mineralization in osteoblast‐like cells. The fabricated 3D scaffolds with tetrakaidecahedral geometry, referred to as nanolattices, had a stiffness range of 0.7–100 MPa with each type of nanolattice under mechanical assays. It was found that 3D scaffolds with Ti‐coated surfaces under optimal microenvironmental conditions may facilitate cell growth, as the stiffness resembles that of cartilage (~0.5–3 MPa).^27^ As described above, relatively lower stiffness may be beneficial for osteogenesis. A recent study reported that by controlling the decalcification time (1 h, 12 h, and 5 days), distinctive compressive modules (0.67 ± 0.14 MPa [low]), (26.90 ± 13.16 MPa [medium]), and (66.06 ± 27.83 MPa [high]) were constructed with demineralized bone matrix scaffolds. Both in vitro experiments with cells and in vivo experiments with subcutaneous implantation in rats indicated that the low scaffolds could promote osteogenesis and bone regeneration.^170^ Although it is widely known that MSCs and osteoblasts respond to both substrate topography and stiffness,^171^ the underlying mechanism still being investigated.

## 6 OTHER ACELLULAR BONE SCAFFOLD MATERIALS

**FIGURE 13:**
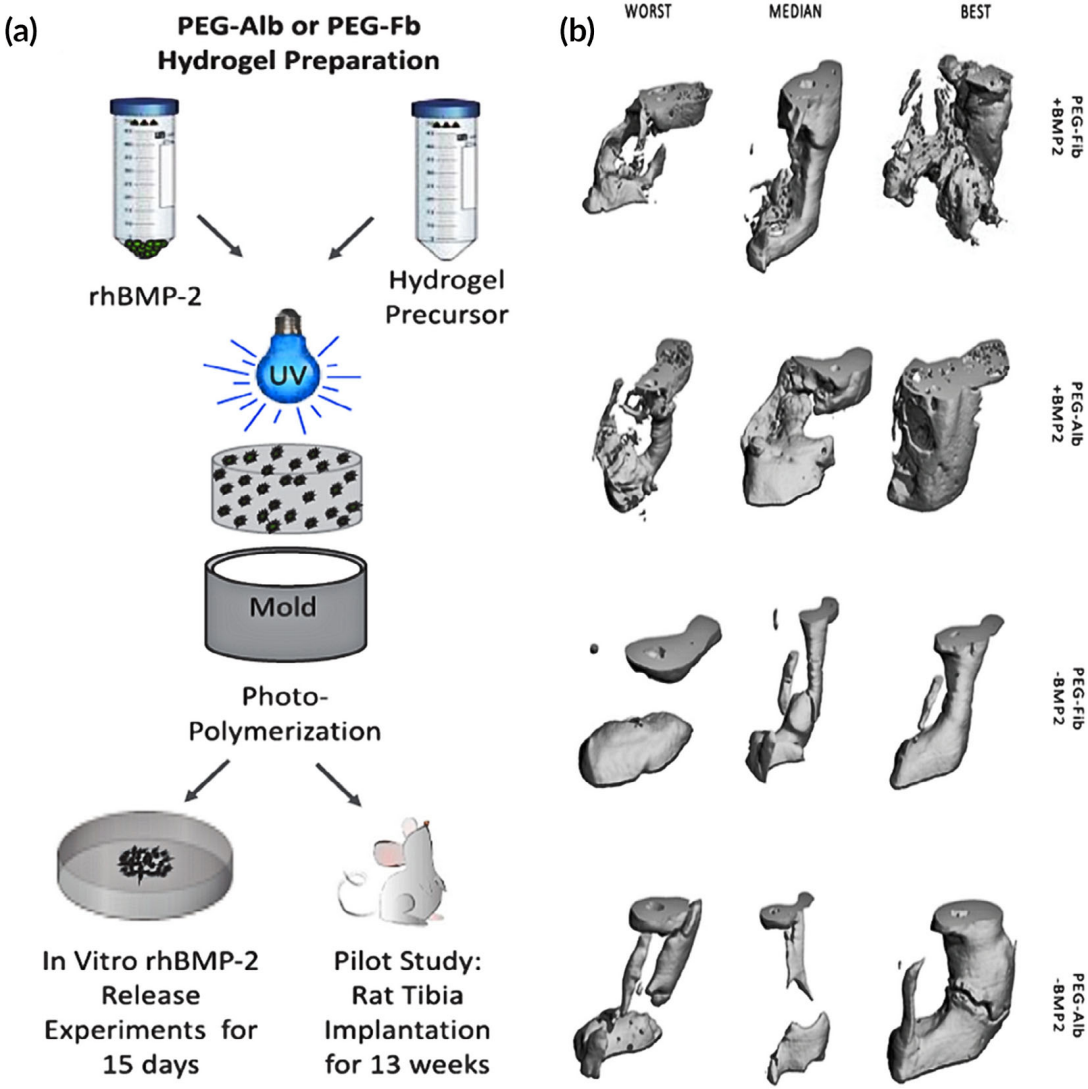
(a) Hydrogels are formed by photopolymerization. The cross‐linked hydrogels containing recombinant human BMP‐2 (rhBMP2) are used for in vitro release studies, swelling and rheological characterizations, or in a rat tibial defect model. (b) μCT imaging of rat hind limbs implanted with hydrogels. All treatments are divided into best, median, and worst; those relating to the new bone formation observed by quantitative μCT of the four treatments *Source*: Reprinted with permission from O. Kossover, N. Cohen, J. Lewis, Y. Berkovitch, E. Peled, & D. Seliktar. Growth factor delivery for the repair of a critical size tibia defect using acellular, biodegradable PEG‐albumin hydrogel implant. *ACS Biomater. Sci. Eng*. 6 (2020) 100–111.^180^ Copyright (2020) American Chemical Society

**FIGURE 14:**
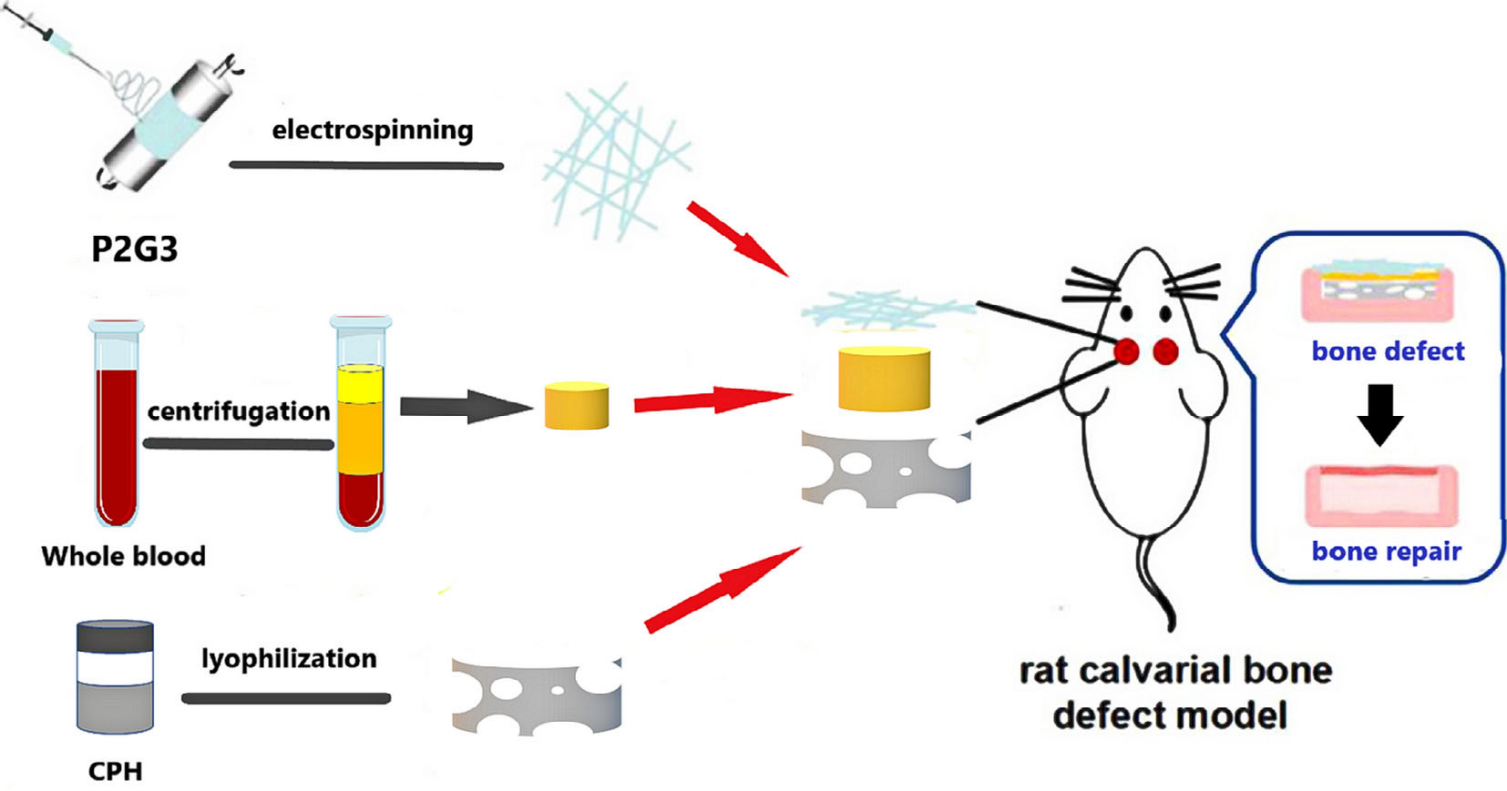
Schematic illustration of the preparation of triple‐layered scaffolds containing P2G3 nanofiber films fabricated by electrospinning, composite CPH hydrogels formed through electrostatic interaction, and platelet‐rich fibrin (PRF) centrifuged from whole blood, as well as their application in vivo

Apart from the strategies of constructing cell‐free scaffolds for bone regeneration described above, other methods will also be discussed, including surface‐immobilized proteins and other biomolecules, utilization of cellular bioenergy, and bone adhesives. All approaches seek to facilitate osteoblast differentiation and bone formation at the graft site, leading to integration between the implant surface and the native bone tissue. The use of seeding cells, mainly MSCs, is one of the main approaches for the construction of cellular scaffolds for bone regeneration but has the disadvantages of potentially damaging healthy tissue as well as substantial costs involved in the isolation and expansion of the cells. Additionally, a comprehensive understanding of stem cells in terms of their terminal differentiation and hypertrophy is lacking, affecting their applications in clinical therapy.^172^ Cell‐free approaches with a surface‐functionalized scaffold can promote bone regeneration by recruiting endogenous osteoblasts to ameliorate bone defect repair. Protein immobilization, first performed on glass substrates, is derived from peptide immobilization. The proteins regarded as enhancing osseointegration can be divided into two groups: (a) ECM proteins that present adhesive sites for cells and (b) proteins that provide signals that activate pathways of bone formation.^173^ Bone morphogenic protein‐2 (BMP‐2) has been found to be beneficial in osteogenesis and bone metabolism^174^ but presented safety concerns for clinical application. Although supraphysiological doses of BMP‐2 may cause adverse effects, including immunological reactions, edema, and heterotopic bone formation,^175^, ^176^ the protein is indispensable for osteogenesis. In order to overcome the limitations of short half‐life and rapid clearance caused by supraphysiological doses, a novel biomaterial vehicle composed of heparin microparticles (HMPs) and alginate hydrogels surrounded by a PCL nanofiber mesh was manufactured. The PCL nanofiber mesh can promote initial tissue and cell filtration into the mesh pores while the functionalized alginate hydrogels act as the carrier delivering HMP and BMP‐2 to facilitate cell attachment and infiltration, enhancing growth factor activity, and hydrolytic degradation.^177^ While the primary limitations are safety and efficacy issues associated with the burst release of BMP‐2, HMP loading may offer a promising strategy to ameliorate the safety of BMP‐2 delivery. A novel BMP‐2 delivery system has been created using the technology of NIR‐responsive hydrogels and inducible transgene expression. This involves the capture of genetically engineered cells in hydrogels which can be activated by an NIR laser inducing the release of BMP‐2 through photoinduced mild hyperthermia. The NIR‐responsive hydrogels encapsulating the cells can express BMP‐2 in the bone defect and can further induce the formation of new bone tissue. As the BMP‐2 is under strict spatiotemporal control, this system offers significant advantages for bone tissue engineering.^178^ Scaffolds manufactured by multiple techniques and comprised of porous Ti‐alloy implants and interconnected channel structures have been created by Teng et al. The microstructure of the implant surface sites provided by microarc oxidation for coprecipitation of the Ca,P layer with BMP‐2 facilitated the sustained release of BMP‐2.^179^ Kossover et al. developed a cell‐free, biodegradable hydrogel graft composed of polyethylene glycol (PEG) and denatured albumin to promote a more sustained BMP‐2 release compared to PEG‐fibrinogen (PEG‐Fib) (Figure 13a). Both hydrogels with rhBMP‐2 had similar effects on bone formation and repair, repairing a 5 mm gap in the tibia simultaneously with hydrogel resorption from the defect site (Figure 13b). Furthermore, rhBMP‐2 exhibited sustained delivery through the biodegradable hydrogel while bone formation coincided with its removal from the defect locale.^180^ In contrast to a single growth factor with potentially limited effects, it is possible that a combination of bioactive factors acting on osteoinduction can not only amplify the effects but possibly also abate adverse effects. Platelet‐rich fibrin (PRF), a platelet concentrate derived from whole blood,^181^ containing a variety of immune cells and growth factors, is cost‐effective and has been popularized in clinical treatment compared with commercially available growth factors. The PRF can provide powerful regeneration stimulation and has potential applications in tissue regeneration, especially bone regeneration. Zhang et al. designed nanofiber films composed of polycaprolactone/gelatin (PG) by electrospinning, acting as barriers against fibrous tissue infiltration into the defect locales; chitosan/poly (γ‐glutamic acid)/hydroxyapatite (CPH) hydrogels were constructed by lyophilization and electrostatic interaction with PRF to induce bone formation via the release of growth factors (Figure 14). The multifunctional composite scaffolds provide barriers, osteoconduction, and the release of bioactive substances, promoting mineralization and bone regeneration in vitro and in vivo, and also provide a novel strategy for clinical bone repair.^182^ The ECM acts as the mechanical support for cells in vivo while matrix‐cell interactions are fundamental in modulating cell activities.^183^ To advance cell viability and function within materials, immobilizing bioactive ligands on 3D scaffolds is essential for interaction with stem cells.^184^, ^185^ Recently, a surface ligand with a specific amino acid sequence, namely the tripeptide arginine‐glycine‐aspartic acid (RGD) motif, has been integrated with transmembrane cell adhesion proteins for improving cellular adhesion to the ECM.^186^ The RGD motif is a cell‐recognizable sequence discovered^182^ in numerous ECM proteins and blood proteins. In this respect, Yassin et al. designed a superior 3D niche, composed of PLLA‐bearing thiol groups for RGDC adhesion and a copolymer of l‐lactide and trimethylene carbonate (TMC), capable of maintaining hBMSC viability and stimulating osteogenic differentiation. By incorporating such copolymers with modified PLLA with thiol groups capable of RGDC attachment and regularly distributed along the polymeric chains, a niche with the ability to promote the interaction between scaffold materials and cells can be constructed. Results indicated that the use of the RGDC‐functionalized scaffolds produced increased ALP activity and upregulated osteocalcin expressed compared to controls.^187^ Furthermore, a cell‐free and growth factor‐free hydrogel that can induce angiogenesis, osteogenesis, and innervation has been created. The formation of new blood vessels is critical for the recovery from tissue damage, especially prior to new bone formation, due to the nutrition and oxygen requirements of growing tissues. However, the role of nervous tissue is poorly described compared to angiogenesis although innervation is necessary for the regeneration and repair of many tissues.^188^ In this respect, Santos et al. formulated a series of hydrogels composed of elastin‐like polypeptides (ELPs), polyethylene glycol (PEG), and different concentrations of the adhesive peptide IKVAV. For the in vitro evaluation of MSC differentiation, a gene expression panel of various osteogenic and angiogenic markers was analyzed, including Runx2, BMP2, OSX, OPN, and VEGF. The results indicated upregulation of all genes with 50% IKVAV in comparison to ELP + PEG. For SNs, with the incorporation of IKVAV, cells incubated with 50% IKVAV showed complex networks of longer neurites compared to the other incubation compositions. However, the same incubation conditions induced angiogenesis and innervation encircling the implant without the presence of inflammation in the subcutaneous graft.^188^ Innate small molecules can be sequestered to regulate various cellular activities in vitro, but using the native small molecules for tissue repair in vivo is reported rarely. Recently, the strategy of sequestration of extracellular adenosin has been showed. By levering the affinity of boronate molecule with adenosine and its transient surge following tissue injury, a synthetic biomaterial showed the sequestration of innate adenosine and the acceleration of bone repair in the fracture site of a murine model.^189^

**FIGURE 15:**
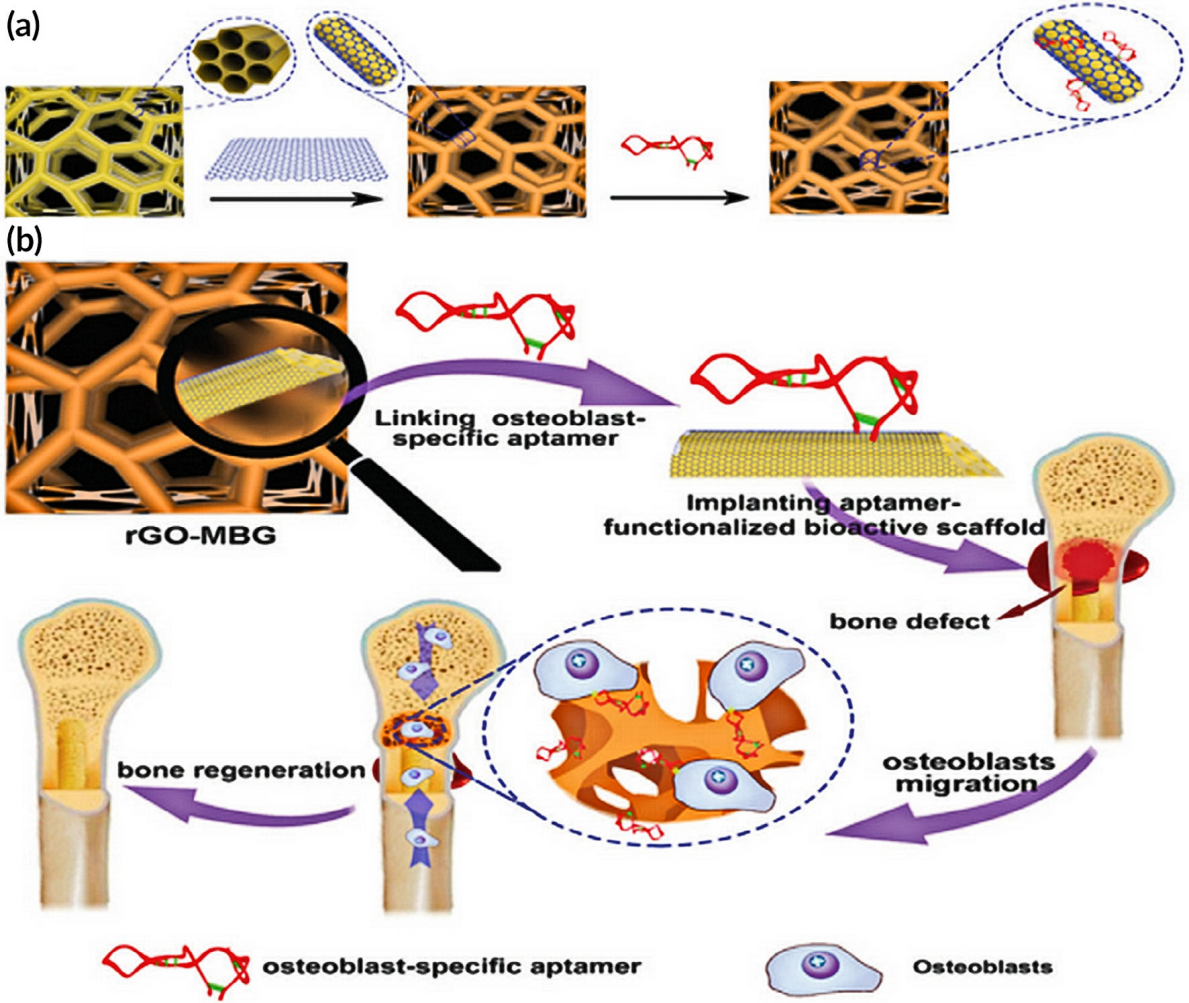
(a) Schematic diagram of scaffold construction. (b) Overview of rGO‐MBG‐AP in critical bone defect repair *Source*: Adapted with permission (Wang et al.^197^)

Graphene, a new allotrope of carbon with a 2D honeycomb lattice, has received great attention in the fields of materials science^190^, ^191^, ^192^ together with its derivatives. With the ability to accelerate differentiation in MSCs and osteoblasts, graphene is attractive for bone tissue engineering.^193^ Furthermore, due to its superior mechanical properties and biocompatibility, graphene has the potential for incorporation with other scaffold materials to intensify their mechanical features and promote bioactivity. Additionally, reduced graphene oxide (rGO), also known as chemically modified graphene, has numerous carboxyl groups on its surface^194^ which can be modified to endow bioactive functionality to recognize specific targets.^195^ For instance, single‐stranded oligonucleotide aptamers that can bind to target molecules with high specificity and affinity.^196^ In this respect, a hierarchically macro‐mesoporous bioactive glass (MBG) with an osteoblast‐specific aptamer and rGO surface coating has been prepared as a novel 3D bioactive porous scaffold (Figure 15a,b). With the combination of improved mechanical strength provided by rGO and osteoblast targeting by the aptamer, the rGO‐MBG‐AP scaffold was found to be superior for bone repair in critical bone defects by recruiting osteoblasts and promoting their differentiation.^197^ Another application of rGO with the incorporation of bioactive peptides was demonstrated by Eckhart et al. They used a synthetic approach of covalent attachment of reduced GO and synthetic peptides, namely Pep‐G. This innovational technique not only provides conjugated peptides that can be precisely defined but also can control for the weight of the peptide molecule. It was found that combining the properties of mechanically robust 3D constructs and electrostatically assembled layer‐by‐layer coatings, the Pep‐G materials provided the conductivity and bioactivity to promote stem cell differentiation when PC12 cells were grown on a p(Lys)_long_‐G pellet with electrical stimulation, showing the enhancement of adhesion in comparison to the CG pellet control.^198^ In addition to the coating of proteins and biomolecules, the surface biosilicification of scaffold materials without exogenous cells and growth factors can also be valuable for bone tissue engineering. Thus, through the process of surface silicification, xenogeneic porcine demineralized cancellous bone (DCB) could be processed into porous nanosilica‐collagen (nSC) scaffolds to promote bone regeneration. In this report, three types of silica precursors were acted for surface biosilicification in situ on collagen scaffolds derived from porcine DCB as a template. Due to the surface functionalization, these nSC scaffolds provided the topographical and chemical cues to produce an osteoinductive microenvironment that could facilitate native MSC recruitment and osteogenesis with a probable underlying mechanism being the abundance of negatively charged silanol groups (Si‐OH) of the nanosilica on the scaffolds that interact with surrounding mineral ions.^199^ In view of both in vitro and in vivo results, the nanosilica‐functionalization scaffolds promoted in situ bone regeneration and, moreover, indicated great potential for the treatment of clinical bone defects.^200^

In addition to immobilization of the above‐mentioned bioactive agents for promoting osteogenesis, combining these with anti‐inflammatory and antibiotic drugs also assists bone repair.^201^ Tetracycline (TC) has been applied widely as an antibacterial agent^202^ due to its well‐known antimicrobial activities. Besides its affinity for Ca^2+^, intermolecular interactions, including Van der Waals forces and hydrogen bonding, between the hydroxyl groups of TC and the apatite, facilitate delivery to the bone.^203^, ^204^ Choosing an appropriate material as carrier is significant not only to prolong the drug release but also to produce additional therapeutic effects. Polyurethane, as a synthetic polymer material, has been shown to enhance new bone formation. The use of polyurethane as an injectable biodegradation scaffold has been found to significantly increase antibiotic release compared to poly(methyl methacrylate) (PMMA) beads, together with facilitating long‐term drug release.^205^ Another report showed that a slower release could be realized through the combination of the porous polyurethane scaffold and decreased water solubility of vancomycin. Due to the reduction of water solubility, the burst release was reduced by the precipitation of the hydrophilic vancomycin hydrochloride.^206^ A novel multifunctional carrier composed of microporous silica nanoparticles and poly(N‐isopropylacrylamide‐b‐(2‐(dimethylamino)ethyl methacrylate) (MSN‐PNI‐PDMA) has been found effective in reducing inflammation and infection and enhancing osteogenesis by loading dexamethasone (DXMS) and an ECM‐derived peptide.^207^ There are various reports on the delivery of antimicrobial compounds within TNTs for bone regeneration in the literature.^208^ Lee et al. reported a variety of bioactive electrospun nanofibrous membrane with immobilized lactoferrin that promoted bone regeneration while modulating inflammation. The results indicated that the combination may be an effective treatment strategy for simultaneously alleviating inflammation and inducing bone repair.^209^ Another scaffold material with anti‐inflammatory and osteogenetic properties has been investigated by Zhang et al. who showed that a tetra‐PEG hydrogel encapsulated with aspirin could promote periodontal ligament stem cell‐mediated bone regeneration through coincubation.^210^

In summary, biological functionalization of scaffold material surfaces not only involving immobilization of proteins, bioactive peptides, and specific aptamers, but also modification of nanosilica, is a broadly investigated field with significant potential for translating into clinical application. Due to cell/GF‐free, one‐step surgery, the functionalization of scaffold surfaces can simultaneously recruit and provide adhesive surfaces for cells to augment their biological responses, further enhancing tissue regeneration.

**FIGURE 16:**
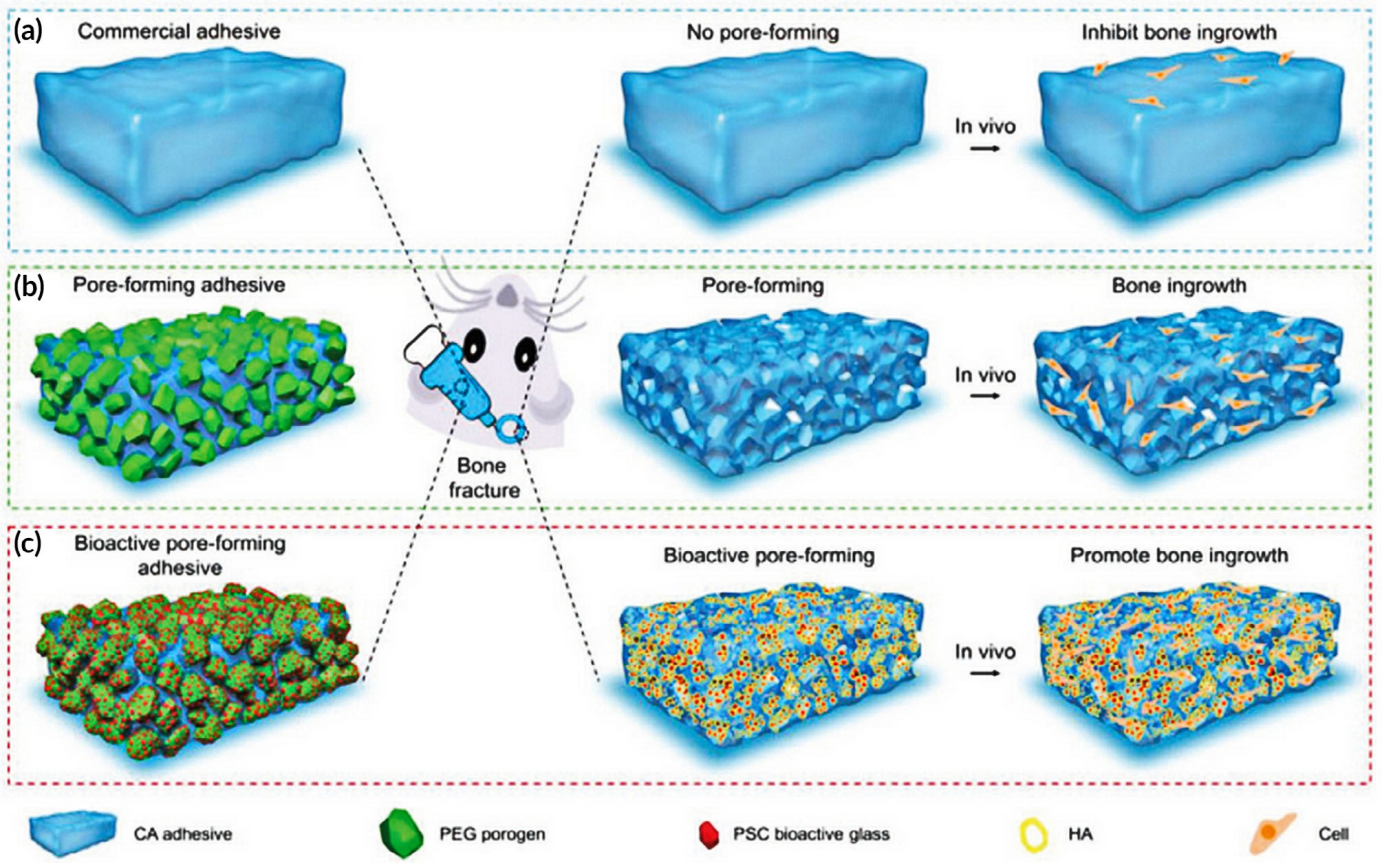
Design of bioactive pore‐forming adhesives. (a) Commercial CA‐based adhesive (blue) has no pores, thus inhibits cell migration and bone healing. (b) Preliminary design of pore‐forming adhesives with encapsulated PEG microparticles (green). Formation of pores by PEG dissolution enables cell replacement and growth. (c) Bioactive pore‐forming adhesives incorporate PSC/PEG composite porogens (red particles are prewrapped PSC bioactive glass). These adhesives can create pores together with a bioactive HA layer (yellow) to further promote bone regeneration *Source*: Reprinted with permission (Xu et al.^215^)

**FIGURE 17:**
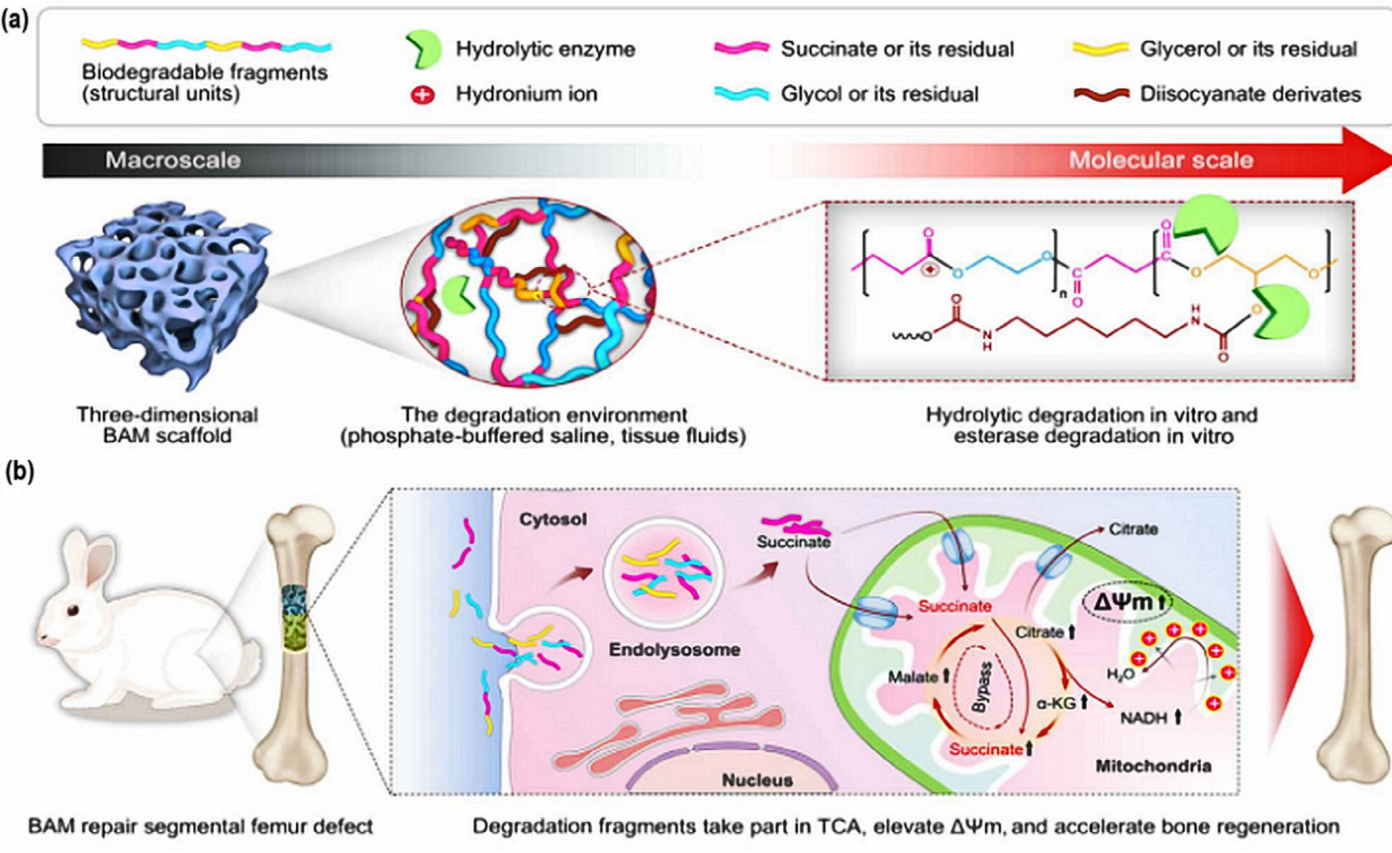
(a) Schematic of the chemical structures and proposed in vitro or in vivo degradation mechanism of bioenergetic‐active material (BAMs). (b) Potential mechanism of degradation fragments mediated bioenergetic effects for enhanced bone regeneration. α‐KG, α‐ketoglutarate; NADH, reduced form of NAD^+^ *Source*: Adapted with permission (Tang et al.^28^)

Instead of the traditional invasive materials for bone fracture repair, these adhesives may revolutionize bone‐implanted surgeries.^211^, ^212^, ^213^ Many adhesives can satisfy biological criteria, such as biocompatibility and cell attachment while the dense layer provided by the adhesives does not cause bone cell ingrowth because of its nondegradability and chemical integrity.^214^ Thus, it is imperative to improve on the advantages of bone adhesives by combining the features of porosity and bioactivity. Adhesives with these properties have been designed by blending PEG porogens with pre‐encapsulated PSC‐BG particles (Figure 16). Through a series of studies in vitro and in vivo, the PSC/PEG/CA adhesive showed the promotion of cell growth, proliferation, and differentiation by the bioactive micrometer‐sized pores in vitro, while it effected stability of the fracture and acceleration of bone regeneration in vivo, due to its strong and rapid‐acting adhesive force. Accordingly, bone adhesives open the prospect of high‐performance biomaterials for clinical application.^215^ In conclusion, the dual functions of bone adhesives, involving robust instant bonding and promotion of bone regeneration, are imperative for the clinical treatment of bone fracture without adverse invasive surgeries. Thus, the bone adhesives represent a novel strategy to provide us with a distinctive direction for bone tissue regeneration. In the field of regenerative medicine applications, tissue implants as well as the delivery of biological factors such as growth factors, have improved stem cell transplantation for many years.^216^, ^217^ However, there have been complications involving tumor development and the formation of ectopic tissue caused by the addition of exogenous cues.^218^ Thus, there is an imperative need for “off‐the‐shelf” materials tunable to present suitable biological features to promote the efficiency of bone regeneration. In mammals, energy metabolism plays an important role in many processes including tissue repair and regeneration. The main source of cellular energy is adenosine triphosphate (ATP). Recently, the development of cellular bioenergetics (CBE) has highlighted the strategies of delivery of bioenergy for treatment purposes.^219^, ^220^ However, long‐term bioenergetic effects for bone repair in complicated tissues still require further research. The bioenergetic scaffold, composed of synthesized poly‐glycerol succinate prepolymers and different concentrations of ethanediol substitutions (PEGS) has suitable degradation features that promote the release of degradation factors. The fragments were internalized through cell endosomes and, following hydrolysis to produce metabolic intermediates, can enter the mitochondria to affect the tricarboxylic acid (TCA) cycle (Figure 17). Through a series of experiments, both in vitro and in vivo results demonstrated that the degradation products instead of retaining the chemical or physical features of the scaffold materials may enhance bone regeneration via bioenergetic metabolism pathways. Moreover, the underlying mechanism of the promotion of repair by BAM may be that the elevated content of extracellular ATP can lead to a signaling cascade for enhanced bone repair, referring to the upregulation of purine receptors and the transduction of downstream signals via c‐Jun at the level of the gene.^28^ In conclusion, the development of bioenergetic materials described here offer an uncomplicated and highly effective way to regulate the energetic demands at the early stages of bone repair. Thus, a novel clinically translated strategy of energy‐related materials engineering has the potential to enhance the treatment of patients with bone disease and other tissue damage.

Although MSCs can successfully mediate healing, the direct application of transplanted MSCs is rare.^221^, ^222^ The importance of paracrine mechanisms in MSC‐mediated processes is now accepted.^223^ MSC‐conditioned media (MSC‐CM) which can be collected in vitro from MSC cultures contain cytokines, chemokines, and growth factors,^224^ as well as extracellular vesicles (EVs).^225^ It has been reported that MSC‐secreted factors are conducive to the regeneration of bone defects, which may perhaps be attributed to the reduction of oxidative stress in aged BMSCs and the reverse of age‐related bone loss.^226^ Moreover, under hypoxia, the enhancement of MSC‐CM‐secreted factors can increase bone healing by simulation of endogenous MSCs.^227^ EVs isolated from MSC‐CM incorporated with bioceramic scaffolds were shown to promote bone regeneration in a dose‐dependent manner in a rat model by activating the P13K/AKt signaling pathway.^228^ In another report, the encapsulation of MSC‐EVs not only promoted bone regeneration but also enhanced angiogenesis, induced by EVs through the upregulation of HIF‐1α and VEGF expression.^229^ However, the preclinical evaluation of MSC‐CM and MSC‐EVs is insufficient to meet the requirements to demonstrate the clinical efficacy of this method. One recent clinical report of bolus CM administration showed that MSC‐CM derived from allogenic BMSCs loaded via biomaterials promoted bone tissue regeneration successfully in eight patients.^230^ Despite the small number of clinical studies, the potential of MSC‐EVs for therapeutic application is based on strong theoretical principles together with increasing preclinical evidence. Although the MSC‐secreted EVs have significant prospects in the tissue regeneration field, many challenges need to be resolved before clinical application, including the choice of optimal MSC source and route of administration, as well as a complete understanding of the bioactive constituents and mechanisms of action.^231^

## 7 PRODUCTION TECHNIQUES FOR CELL‐FREE BIOMIMETIC SCAFFOLDS

The manufacture of acellular biomimetic scaffolds for bone regeneration, including traditional and free‐form technologies, have been investigated in novel regenerative tissue engineering. We have previously probed the characteristics and applications of cell‐free scaffolds; here, we investigate the manufacture techniques of both conventional and novel bone scaffolds.

### 7.1 The fabrication of ion‐functionalized scaffolds

Inorganic ions with the capability of rapid dispersion through the cellular membrane and regulation of different cellular activities have been extensively studied.^35^, ^232^ Because of the different properties of the various ions, distinct encapsulation techniques allowing specific spatial and temporal release to the damaged sites have been investigated.^233^ The inherently stable nature of these ions allows them to be used in various ways. Freeze‐drying, as a traditional production technique, is commonly used.^7^, ^39^, ^41^, ^46^ This can lead to the formation of solvent ice crystals surrounded by polymer aggregates. When the solvent undergoes direct sublimation from the solid phase into gas, an interconnected porous structure emerges within the dry polymer scaffold. Apart from lyophilization, solid‐state sintering^9^ and the microemulsion‐assisted sol–gel approach^10^ have also been used in the manufacture of biomimetic ion‐functionalized scaffolds.

### 7.2 The manufacture of decellularized extracellular matrix scaffolds

To maintain the original tissue's bioactivity, the process of decellularization must prevent the loss of the original ECM components while removing other cellular proteins and nucleic acids. If the latter components of the decellularized tissue have not been completely removed, an immune reaction may occur in the host after implantation,^234^, ^235^ resulting in inappropriate tissue remodeling and limiting the regenerative potential of the decellularized tissue.^236^ Preserving the integrity of the structure of the dECM components is the key point for various decellularization methods, including physical, enzymatic, and chemical processes, as well as the combination of two or three approaches.

Freeze–thaw and osmotic pressure procedures used in physical decellularization can cause cell lysis without critical disruption of the structure of the original tissue. The formation of ice crystals can penetrate cell membranes during freezing and thawing, which can be repeated multiple times during the process. With osmotic lysis, either hypertonic^237^ or hypotonic solutions can disrupt the plasma membrane through osmotic shock. Other physical decellularization approaches include the use of hydrostatic pressure,^238^ sonication,^239^ and electroporation.^240^

However, physical decellularization is the mildest decellularization method, leaving most of the ECM components and structures undamaged^241^ and resulting in incomplete removal of cellular fragments from the original tissue. Therefore, a combination of two or three decellularization methods may be more advantageous than a single method. Boram et al. demonstrated two decellularization methods for ECM deposited scaffolds: one involving physical decellularization with three freeze–thaw cycles in liquid nitrogen and a 37°C water bath, respectively, and the other a chemical method with SDS solution immersion.^13^ Wang et al. described the manufacture of C/G/A‐dECM scaffolds with three decellularization methods, including freeze–thaw cycles, immersion in trypsin/EDTA and isopropanol, and treatment with DNase I and RNase A.^12^ Repeated freezing and thawing combined with enzymatic methods were used for the decellularization of PRF,^78^ similar to those described by Wang et al. Furthermore, Bianco et al. reported a novel decellularized bone marrow scaffold produced by a detergent‐free protocol with mechanical rupture, enzymatic treatment, and polar solvent extraction.^242^

Acidic or basic conditions and detergents are considered as the two chemical methods of decellularization. Treatment of tissues with acids or bases results in cell degradation and the elimination of cellular components such as nucleic acids. However, as exposure to bases can result in the critical loss of GAGs,^243^, ^244^ this treatment is rarely considered as an option for the decellularization of cartilage and bone tissue. Another chemical decellularization method involves the use of detergents, which fall into three categories: nonionic, ionic, and zwitterionic. Triton X‐100, as one of the nonionic detergents, lyses cells by insertion into the lipid bilayer, rupturing the cell membrane. Although destroying the lipid bilayer, the protein–protein interactions are retained^245^ as their native structure remains intact. Additionally, the ionic detergent sodium dodecyl sulfate (SDS) not only ruptures the cell membrane but completely denatures the proteins. Just as everything has two sides, ionic detergents are commonly considered harsher than nonionic detergents, which are more conducive to the retention of the ECM structure. Zwitterionic detergents are relatively mild for tissue decellularization, and one of the zwitterionic detergents, 3‐([3‐chola midopropyl] dimethylammonio)‐1‐propanesulfonate (CHAPS) shows properties of both an ionic and nonionic detergent.^246^ Therefore, compared to their ionic counterparts, zwitterionic detergents cause less protein denaturation and less removal of cellular components.^247^, ^248^

To add a beautiful thing to a contrasting beautiful thing, enzymatic decellularization is often applied after chemical decellularization, leading to further cellular degradation and the elimination of remaining nuclear components from the original tissue. Two classes of enzymes, proteases and nucleases, are most commonly used. Proteases such as trypsin hydrolyze peptide bonds. Treatment with trypsin can significantly rupture ECM proteins including elastin and collagen.^242^ Furthermore, combined with chelating agents such as ethylenediamine‐tetraacetic acid (EDTA), enzymatic approaches can disrupt cell adhesion to ECM proteins by removal of ions such as calcium.^246^

### 7.3 The manufacture of biomimetic scaffolds with micro/nano‐structural features

There are diverse methods for the construction of biomimetic scaffolds with nano/micro‐structural characteristics. The modification of surfaces and interfaces has a significant influence on the biofunctions of scaffold implants in vivo. Metal implants with modification of micro/nano‐topography can simulate the hierarchical structure of bone tissues to a certain extent, and, furthermore, can regulate cell activities including migration, proliferation, differentiation, and, ultimately osteogenesis and osseointegration in vivo.^249^, ^250^, ^251^ Hydrothermal treatment is often regarded as a method for the construction of a nanostructured surface. Xia et al. reported the construction of nanostructured HAp bioceramic scaffolds with three kinds of surfaces by hydrothermal reactions: nanosheet, nanorod, and nanorod and microrod hybrids. The effects of these three surface topographies on attachment, proliferation, and osteogenic differentiation of BMSCs and ASCs, as well as related mechanisms, were systematically investigated.^85^, ^86^ The combination of nanostructures and silicon‐substitution on hydroxyapatite for bone regeneration has also been demonstrated. The calcium silicate used as raw material was produced by sintering chemically precipitated CaS powders, and, furthermore, Si‐substituted HAp scaffolds were also produced through hydrothermal reactions into nanosheet and nanorod surfaces.^89^ Zhao et al. designed HAp bioceramics with ordered micropatterned surfaces, which were manufactured via a layer of ordered micropatterned nylon sieves as templates. Prior to the addition of the ordered micropatterned surfaces, the HAp nanoparticles were synthesized by a wet chemical precipitation method and subsequently added via calcination.^88^

For the creation of unidirectional pores structures, Wu et al. reported the manufacture of gelatin‐strontium‐substituted calcium phosphate scaffolds. This involved two steps, including coprecipitation in a gelatin solution followed by the manufacture of oriented microtubular structures using a freeze‐drying technique.^93^ A 3D microtubule‐orientated PLGA scaffold has been constructed based on a phase‐separation method. Specifically, the polymer solution was first poured into a mold and the mold was cooled from bottom to top until reaching −20°C, inducing solid–liquid phase separation. After complete crystallization, the phase‐separated and solidified polymer/solvent systems were freeze‐dried, and, finally, the oriented PLGA scaffold was completed.^94^, ^95^

In contrast to the unidirectional pore structure, radially directed pores have better architectural stability and promote stronger interactions between the new tissues within the scaffold and the surrounding native tissues. Dai et al. reported the production of acellular HA‐MA/PLGA scaffolds via controlled directional cooling of HA‐MA solutions and lyophilization of the hybrid scaffold of dry O‐HA‐MA scaffolds and PLGA solutions.^96^ As another radially‐aligned scaffold, fibrous material scaffolds coated with polydopamine have been created by electrospinning to guide directional migration of MSCs.^97^

It is relatively complicated to repair the bone defect with irregular shapes and hierarchical structures that represent a combination of soft and hard tissues. A hierarchically structured scaffold for repair of tendon‐to‐bone has been designed and manufactured. Three regions of the scaffold were arranged as follows: collagen fibers are deposited on the tendon side, composed of an array of channels and alignments, the middle region is regarded as a region of stress transfer and has a mineral gradient, while the bone side is a mineralized inverse opal that promotes the integration of the scaffold with the bone. In terms of the manufacturing process, first, the HAp gradient was created by layer‐by‐layer coating, then the HAp/PLGA‐coated scaffolds were machined through a CO_2_ laser, and, finally, the composite scaffolds were completed by removing the opaline lattice which acted as a sacrifice template.^98^ Due to the shape memory effect, chitosan can be utilized to construct various porous scaffolds for repairing irregular bone defects.^109^ Wang et al. reported 3D superelastic, flexible scaffolds composed of SiO_2_ NF–CS and SiO_2_–CaO NF/CS, respectively. Both composite scaffolds were fabricated by sol–gel electrospinning, followed by a lyophilization technique.^110^, ^111^ Grottkau et al. reported the creation of anatomically shaped bone scaffolds using 3D printing molds as well as PLA and PLA‐HA casting and salt leaching. This technique is a superior tool in constructing personalized, patient‐specific bone graft scaffolds with various excellent characteristics.^252^

### 7.4 The manufacture of physical stimuli‐responsive bone scaffolds

Photothermal therapy (PTT) has been reported to have wide applications due to its dual functions of eliminating tumors while stimulating tissue regeneration. Recently, bioactive glass (BG) scaffolds functionalized by CuFeSe_2_ nanocrystals (BG‐CFS) have been prepared by the solvothermal method with the 3D printing technique. Along with the solvothermal response processing, the surface of the BG scaffolds can be spread with CuCuFeSe_2_ nanocrystals which endow the BG scaffolds with superior photothermal performance. Furthermore, the composite scaffolds have the capacity of stimulating osteogenic gene expression in BMSCs and ultimately promoting regeneration in the bone defect.^114^ Other bifunctional scaffolds have also been reported, including the fabrication of a 3D‐printed bioceramic scaffold with a uniformly self‐assembled Ca‐P/polydopamine nanolayer surface, which is both biocompatible and biodegradable, as well as incorporating the superior photothermal effects of polydopamine.^132^ The production of MoS_2_ nanosheets and AKT bioceramic scaffolds via a 3D printing method and a hydrothermal approach has also been reported.^115^ Ma et al. reported the fabrication of novel multifunctional scaffolds comprised of nHA/GO/CS. Among these materials, GO powders were synthesized through a modified Hummer's method, following which the GO/nHA was dispersed in deionized water mixed with the CS scaffolds and subsequently lyophilized.^30^ Utilizing the highly efficient NIR photothermal effect, the BPs‐PLGA scaffolds with complete biodegradation could facilitate osteogenesis in vitro and in vivo. In brief, the BPs were prepared via solvent exfoliation of bulk BP crystals, after which the BPs‐PLGA were made by mixing the BPs and PLGA, followed by slow evaporation of the solvent.^116^ Besides these bifunctional photothermal scaffolds an innovative thermodynamically controlled architecture, termed hierarchical intrafibrillarly mineralized collagen (HIMC), has been constructed, in which the self‐assembly involved two steps: (a) the fabrication of a high‐energy polyacrylic acid‐calcium (PAA‐Ca) intermediate, and (b) the drive of an energetically downhill process for selective mineralization in the collagenous gap regions. Finally, the synthesized mineralized collagens were lyophilized to form 3D sponge‐like scaffolds.^29^

Tissue regeneration and the regulation of cellular activity through an exogenous and noninvasive method, especially via the construction of cell‐free biomimetic bone scaffolds, have the means of revolutionizing the field of tissue engineering. The application of electric and magnetic fields has gained critical interest due to their advantageous effects on cell adhesion, proliferation, and differentiation in vitro, as well as osteogenesis in vivo. Recently, 3D electromagnetic inverse opal scaffolds with the capacity of generating localized electric fields have been devised. First, the gelatin microspheres were prepared using a microfluidic device, followed by the assembly of the CFO@BFO core‐shell nanoparticles by a two‐step method including hydrothermal synthesis of the CFO core and subsequently sol–gel synthesis of the BFO shell. Finally, the electromagnetic inverse opal scaffolds were constructed by infiltration of the assembled gelatin template with the CFO@BFO/PLLA dispersion under vacuum.^19^ A nanocomposite membrane imitating the endogenous electric potential was prepared and the efficiency of the bone defect repair investigated. The synthesis procedures involved the coating of polydopamine on BaTiO_3_ nanoparticles (BTO NPs), followed by homogeneous distribution of Dopa@BTO NPs in a poly(vinylidene fluoridetrifluoroethylene) (P(VDF‐TrFE)) matrix.^23^ Magnetic biomaterials possessing magnetic properties through the incorporation of magnetic iron oxide particles have been widely described. The approaches for the incorporation of magnetic particles into biomaterials involve blending, doping, in situ precipitation, and the “grafting onto” approach.^253^ Zhang et al. reported the construction of 3D magnetic Fe_3_O_4_ nanoparticles combining mesoporous bioactive glass/polycaprolactone (Fe_3_O_4_/MBG/PCL) composite scaffolds. Through using nonionic block copolymer EO_20_PO_70_EO_20_ (P123) as a structure‐directing agent, the MBG powders were prepared, then, utilizing the coprecipitation approach with some modifications, magnetic Fe_3_O_4_ NPs were synthesized. Finally, the composite scaffolds of Fe_3_O_4_/MBG/PCL were created by a 3D printing technique.^153^ Novel magnetoactive 3D porous scaffolds, comprised of poly(vinylidene fluoride) (PVDP) and magnetostrictive particles of CoFe_2_O_4_, have been prepared. Through the overlapping of nylon templates structures with different fiber sizes, the solvent casting approach has been utilized for constructing different pore sizes.^254^ For other bioinspired composite scaffolds, first, nHA, Fe_3_O_4_ NPs, and CS/COL were prepared and the mixture of the four materials was subjected to in situ crystallization followed by freeze‐drying to produce the porous 3D scaffolds.^255^

Over the last decade, many researchers have observed that the application of external forces can activate MSC osteogenic signaling pathways involving *Runx*2 and *Wnt*. However, current studies have focused on the significant role of internal strength, referring to the importance of cell‐matrix interactions in MSCs. Furthermore, both external mechanical forces and the interaction between resident cells and the matrix can induce MSC mechanobiology and lineage specification. Several years ago, the effects of substrate alignment and mechanical stimuli on MSC differentiation were reported. Scaffolds composed of nanofiber PLGA were synthesized by electrospinning and the MSC reaction to a combination of cell‐matrix interactions and mechanical stimuli was investigated.^166^ Maggi et al. reported 3D nanoarchitected scaffolds with controllable stiffness that were fabricated via two‐photon lithography (TPL) direct laser writing. The geometrical shape, referring to the nanolattices, was coated with thin conformal layers of Ti or W and the outer layer with TiO_2_. The mechanosensitive reaction of osteoblast‐like cells was then investigated through the track of mineral secretions and the concentrations of f‐actin and vinculin.^27^ In another study, 3D demineralized bone scaffolds with different mechanical properties were constructed through decalcification using EDTA‐2Na for different lengths of time. These scaffolds with distinctive compressive modules were then investigated in cell experiments and animal studies.^170^

### 7.5 The manufacture of other acellular bone scaffolds

Considering the specific cell‐free bone scaffolds for bone regeneration, we will first discuss the immobilization of growth factors. Fibronectin (FN) domains have been used to bind growth factors. Specifically, a recombinant fragment of FN containing three fibrin‐binding sequences, was first produced, followed by the binding of three growth factors, VEGF‐A165, PDGF‐BB, and BMP‐2, to the domains for accelerating skin repair and bone repair.^217^ Kossover et al. reported a strategy of growth factor delivery within poly(ethylene glycol) (PEG) and fibrinogen hydrogels (PEG‐Fib) which were constructed with PEGylated denatured fibrinogen and additional PEG‐DA. PEG‐Alb hydrogels were constructed with PEGylated albumin with additional PEG‐DA. The hydrogels were first mixed with photoinitiator stock solution, then cross‐linked with rhBMP2, followed by exposure to UV light. The rhBMP2‐containingd hydrogels can promote the bridging of bone defects through rhBMP2 delivery.^180^ PRF, comprising abundant growth factors and immune cells can be engineered onto hydrogels for bone regeneration. Triple‐layered composite scaffolds were constructed with polycaprolactone/gelatin nanofiber films as a barrier layer by electrospinning while chitosan/poly(γ‐glutamic acid)hydroxyapatite hydrogels were used as the osteoconduction layer through electrostatic interactions and lyophilization, with PRF as the acceleration cue for bone repair through its combination with composite scaffolds.^182^ Liu et al. reported a 3D biomimetic scaffold constructed with PCL/HA further modified with VEGF. First, the PCL/HA composite microspheres were constructed through an emulsification solvent evaporation approach, then, the microsphere‐based porous scaffolds were prepared by selective lasersintering (SLS). Finally, the surface of the composite scaffold was modified with VEGF165 labeled with fluorescent RBITC for visualizing the vascularized bone regeneration in vivo.^256^

The use of surface‐immobilized proteins and other biomolecules is critical for the development of acellular scaffolds as the scaffolds can be tailor‐made for specific needs. Recently, a porous scaffold prepared with poly(LA‐*co*‐TMC) and poly[(PDS‐LA)‐*co*‐LA] by salt leaching was used to induce osteogenic differentiation. Furthermore, covalent attachment of the adhesion‐mediating RGDC peptide to scaffolds promoted the differentiation of stem cells.^187^ An rGO‐coated bioglass scaffold integrated with an osteoblast‐specific aptamer was manufactured. First, porous bioactive glass sol was prepared by an evaporation‐induced self‐assembly procedure, then, after the preparation and purification of GO, the MBG scaffolds were soaked in the GO/ascorbic acid suspension, followed by heat treatment to reduce the GO which can be coated on MBG scaffold. Finally, the osteoblast‐specific aptamer was added to the rGO‐MBG scaffold and reacted in a reciprocating oscillator for conjugation.^197^ Zhang et al. reported the construction of a biomimetic structure with bio‐surface‐coated Ti scaffolds. The Si‐doped CaP composite scaffolds, utilizing sugar spheres as fore‐forming agents, were coated with vancomycin‐loaded polydopamine‐modified albumin nanoparticles, and cell adhesion‐mediating peptides. First, the sugar spheres acting as pore‐forming agents were synthesized by emulsification, and the porous Ti scaffolds were constructed by the sol–gel approach and sugar sphere template leaching procedure with the Si‐substituted apatite coating processed by incubation in simulated body fluid. The vancomycin‐loaded NPs were prepared via a modified desolvation approach based on bovine serum albumin. In the final step, a thin pDA film was first prepared on the surface of the composite Ti scaffolds, followed by immersion in Van‐pBNPs and soaking in the GFOGER peptide solution. The composite Van‐pBNPs/pep@pSiCaP‐Ti scaffolds were then used for subsequent experiments.^257^

Fracture fixation and bone repair are clinically challenging due to the lack of appropriate materials and adequate fixation strategies. Recently, the use of adhesives has led to the realization of these goals. A novel class of chemical compounds has been derived from dental resin composites and self‐etch primers with the adhesives being methodically designed and manufactured using visible light thiol‐ene coupling. Moreover, the precision of the adhesive strength was completed through fiber‐reinforced adhesive patch methods.^211^ Xu et al. reported a bone adhesive with a pore structure that was superior to commercial bone adhesives which may not adequately support cell infiltration. First, PEG and PEG/PSC porogens were prepared by heat‐melting, grinding, and sieving. Then, the initial PEG/CA pore‐forming adhesives were prepared through the incorporation of the PEG porogens into the mixture containing CA monomers and the PTSA stabilizer. Furthermore, the bioactive PSC/PEG/CA pore‐forming adhesives were constructed by the incorporation of PSC/PEG composite porogens with prewrapped PSC bioactive glass.^215^ Table 1 shows the manufacture techniques for different scaffold materials.

**TABLE 1:** Manufacture techniques for different scaffold materials

Scaffold material	Fabrication technique	Reference
Sr/BG‐G/nHAp	Freeze‐drying	Oryan et al.^7^
SrHAp/CS		Lei et al.^39^
Sr/MgP bioceramics	Solid state sintering	Sarkar et al.^9^
Zn‐MBGNs	Microemulsion‐assisted sol–gel	Neščáková et al.^10^
dECM/BCP	Freeze–thaw/SDS solution immersion	Kim et al.^13^
CS/G/A‐dECM	Freeze–thaw cycles/immersion of trypsin/EDTA/isopropanol/treatment of nuclease	Wang et al.^12^
DeBM	Mechanical rupture/enzymatic treatment/polar solvent extraction	Bianco et al.^242^
Hydroxyapatite (HAp) bioceramic scaffolds with nanosheet, nanorod, and micro/nano‐hybrid surface topographies	Hydrothermal reaction	Xia et al.^85^, ^86^
Si‐substituted HAp		Xia et al.^89^
HAp bioceramics with ordered micropatterned surfaces	Ordered micropatterned nylon sieve/wet chemical precipitation/calcination	Zhao et al.^88^
G/Sr substituted CaP	Coprecipitation/freeze‐drying	Wu et al.^93^
MOIP‐PLGA	Thermal‐induced phase separation	Shen et al.^94^
HA‐MA/PLGA	Controlled directional cooling/lyophilization	Dai et al.^96^
RAFSs	Electrospinning	Shin et al.^97^
SiO_2_ NF–CS	Sol–gel electrospinning/lyophilization	Wang et al.^110^, ^111^
SiO_2_–CaO NF/CS		
PLA/PLA‐HA	3D printing/casting/salt leaching	Grottkau et al.^252^
BG‐CFS	Solvothermal method/3D printing	Dang et al.^114^
Ca‐P/polydopamine nanolayer surface	3D printing	Ma et al.^132^
MoS_2_ nanosheets and AKT bioceramic	3D printing/hydrothermal method	Wang et al.^115^
nHA/GO/CS	Modified Hummer's method/lyophilization	Ma et al.^30^
BPs‐PLGA	Solvent exfoliation/solvent evaporation	Tong et al.^116^
Hierarchical intrafibrillarly mineralized collagen (HIMC)	Two steps self‐assembly	Liu et al.^29^
CFO@BFO/PLLA	Microfluidic device/hydrothermal/sol–gel	Mushtaq et al.^19^
Fe3O4/MBG/PCL	Coprecipitation/3D printing	Zhang et al.^153^
PVDP/CoFe_2_O_4_	Solvent casting	Fernandes et al.^254^
nHA/Fe3O4 NPs‐CS/COL	Crystallization/freeze‐drying	Zhao et al.^255^
Ti/W/TiO2	Two‐photon lithography (TPL) direct laser writing	Maggi et al.^27^
Porine demineralized bone matrix scaffolds	Decalcification	Hu et al.^169^
rhBMP‐2/PEG‐Fib/PEG‐Alb	PEGylated denatured fibrinogen/PEGylated albumin/UV light	Kossover et al.^180^
PCL/G nanofiber‐CS/P/HA‐PRF	Electrospinning/electrostatic interaction/lyophilization	Zhang et al.^182^
PCL/HA/VEGF	Emulsification solvent evaporation/selective lasersintering (SLS)	Liu et al.^256^
Poly(LA‐*co*‐TMC)/poly [(PDS‐LA)‐*co*‐LA]‐RGDC peptide	Salt leaching/covalent attachment	Yassin et al.^187^
rGO/BG/osteoblast‐specific aptamer	Evaporation‐induced self‐assembly/heat‐treating/reciprocating oscillation	Wang et al.^197^
Van‐pBNPs/pep@pSiCaP‐Ti	Emulsification/sol–gel/sugar sphere template leaching/modified desolvation/soaking	Zhang et al.^257^
PSC‐BG/PEG/CA adhesives	Incorporation of PSC‐BG/PEG composite porogens	Xu et al.^215^

## 8 PRESENT CLINICAL STRATEGIES FOR BONE REPAIR AND CHALLENGES TO CLINICAL TRANSLATION

Autologous, allogeneic, or xenogeneic bone grafts, together with synthetic biomaterials, are regarded as the current available methods in clinical treatment.

The iliac crest, as main source of autografts, is the preferred harvesting site. The cancellous bone can be collected intraoperatively and used for preparing bone blocks or bone chips for the filling of bone defects.^258^ To overcome the problem of vascularization, a vascularization cortical autograft has been produced to reconstruct large bone defects.^259^ The applications of a free fibula flap in mandibular and maxillary reconstruction have showed a superior graft survival ratio.^260^, ^261^ Bone allografts, as a substitute for autografts, are commonly harvested from living donors or cadavers, followed by processing and, finally, transplantation into another patient^262^; these are useful due to their easy availability in different sizes and shapes.^263^ Besides the application of allografts alone, their combination with autologous concentrated bone marrow cells also has been reported.^264^, ^265^, ^266^ Xenografts harvested from different species have been reported in clinical treatment; these are advantageous due to availability and their compatible porosity for bone tissue growth and similar mechanical properties to native bone. Karalashvili et al. reported a case utilizing decelluarized bovine bone to repair a large bone defect which showed long‐term retention of the graft shape without resorption and bone integration.^267^ The bovine cancellous bone also played a significant role in the management of tibial fractures in elderly patients, showing good healing results.^268^ Nevertheless, the use of xenografts has been hampered by issues such as graft rejection and failure of tissue integration.^269^, ^270^, ^271^, ^272^

Apart from autologous, allogeneic, and xenogeneic grafts, synthetic scaffolds have been extensively used. HAp scaffolds with loaded MSCs showed notable osteogenic ability with no adverse responses after tumor curettage.^273^ Interconnected porous calcium hydroxyapatite loaded with bone marrow mononuclear cells was effective in the repair of osteonecrosis and avoided collapse.^274^ Comparing the efficacy and safety between HAp/collagen and β‐TCP, the former showed superior bone repair capability but with a higher incidence of untoward effects.^275^ Combining the β‐TCP scaffolds with MSCs also has been reported; these showed more trabecular remodeling in clinical femoral defects with the addition of MSCs.^276^ Furthermore, the application of BoneSave, a commercial bone graft substitute composed of β‐TCP and HAp ceramic, has been investigated. The successful fusion of posterolateral intertransverse spinal^277^ and effective treatment with loss of the acetabulum^278^ has been shown. Calcium phosphate cement produced at ambient temperatures from hydrolysis is distinct from CaP ceramics. These cements are commonly used as fillers via injection or as scaffolds by 3D printing^279^, ^280^; however, due to slow degradation, delayed bone repair occurred.^281^ The use of 3D printing, especially 3D printed ceramic scaffolds^282^, ^283^, ^284^ is able to mimic the microarchitecture and sophisticated anatomical structures of the patient's anatomy.^285^ However, the potential challenges may hamper the translation of 3D printing bioceramics to clinical treatment. The information related to clinical trials and results were added in Table 2.

**TABLE 2:** Clinical trials of scaffold materials for bone regeneration

Scaffold	Trials	Results	Case (*n* = sample)
HAp	MSCs obtained from each patient's bone marrow cells were forced to differentiate into osteoblasts followed by bone matrix formation on HAp ceramics to heal bone tumors using tissue‐engineered implants. Serial plain radiographs and computed tomography images were used to observe results	The strong osteogenic ability of the implants, as evidenced by high osteoblastic activity, was confirmed. The tissue‐engineered HAp was used to fill the patient's bone cavity after tumor curettage. Immediate healing potential was found and no adverse reactions were noted in these patients	Bone tumors (*n* = 3)
IP‐CHA	We have investigated the effectiveness of the transplantation of BMMNCs and cell‐free with IP‐CHA on early bone repair for osteonecrosis of the femoral head	In the BMMNC group, a reduction in the size of the osteonecrotic lesion was observed subsequent to hypertrophy of the bone in the transition zone and three patients were detected extensive collapse. In the control group, severe collapse of the femoral head occurred in six of eight hips	22 patients (*n* = 30 hips) who used BMMNCs with IP‐CHA and 8 patients (*n* = 9 hips) with cell‐free IP‐CHA of osteonecrosis of the femoral head
HAp/type I collagen composite scaffold	The efficacy and safety of HAp/Col were assessed in comparison β‐TCP. X‐ray images and blood tests and observation of the surgical site were performed to evaluate the efficacy and safety of the implants	The highest grade of bone regeneration was more frequent in the porous HAp/Col group than in the porous β‐TCP group (*p* = 0.0004 and 0.0254, respectively). The incidence of adverse effects was higher in the porous HAp/Col group than in the β‐TCP group	Bone defects by benign bone tumors with HAp/Col (*n* = 63) and β‐TCP (*n* = 63)
β‐TCP scaffold	Compare healing quality of implantation into femoral defects during revision total hip arthroplasty, containing either expanded autologous MSC (trial group) or Β‐phosphate alone (control group)	A significant difference in the bone defect healing was observed between both groups of patients (*p* < 0.05). Trabecular remodeling was found in all nine patients in the trial group, and only 1 patient in the control group	Femoral defects with autologous MSC/β‐TCP(*n* = 9) and β‐TCP (*n* = 9)
BoneSave (TCP/HAp)	Analogue scales for pain, patient global impression of change, work status, persisting symptoms and patient satisfaction data, radiological evaluation of fusion was carried out from the most recent spinal radiographs available for each patient	Significant postoperative improvements were seen across all outcome measures in the large majority of cases. Successful fusion was achieved in 56.7% of cases	Posterolateral inter‐transverse spinal defects (*n* = 45)
CGF fibrin/Bio‐oss	Design a clinical trial composed of patients with jaw defects, concentrated growth factor fibrin/Bio‐Oss bone powder was the test group; Bio‐Oss bone powder alone was the control group. Bone alkaline phosphatase (BAP), osteocalcin, and bone mineral density levels were measured, regular examinations and computed tomography scans were also performed in the follow‐up period	The BAP and osteocalcin levels had increased at 1 and 12 weeks postoperatively in both groups. Furthermore, the BAP and osteocalcin levels in the test group were significantly greater than those in the control group at 1 and 12 weeks postoperatively (*p* < 0.05 for all). The bone mineral density in the bone defect area of the test group was also significantly greater than that of the control group at 6 months postoperatively (*p* < 0.05). Evaluation of the regular radiographic scans revealed that the effects in the test group were better than those in the control group	Jaw defects with test groups (*n* = 20)and control (*n* = 20)
Bovine‐DBM/LLLT	A clinical case reported the safe and positive outcome of low level laser therapy in conjunction with demineralized bone matrix of bovine origin in the surgical treatment of a periodontal infra bony defect	By radiological measurement LLLT + DBM showed good results in clinical insertion level (CAL) gain of 4 mm, linear bone gain of 2.5 mm, bone filling of 37% and reduction of defect angle from 68° to 32°, showing a positive treatment result. Safe treatment to approach periodontal regeneration	Moderate chronic periodontitis with chronic localized periodontal abscess at 44 and 45(*n* = 1)

Although a great deal of research has been published on bone tissue regeneration, a few factors still hinder the translation from basic research to the clinic. These include the following scientific and technological challenges: (a) The most appropriate cell type for use in bone regenerative therapy is still unclear. Although mesenchymal stem cells have been applied clinically and experimentally, risks remain.^286^, ^287^ Embryonic (ESCs) and induced‐pluripotent stem cells are indispensable in adult tissue repair and in addition, still require further laboratory manipulation, (b) Sufficient vascularization is required for the survival of the cells and the subsequent bone repair. However, infiltration of neovessels often lacks depth of penetration, limiting the size of viable bone constructs that can be implanted,^288^, ^289^ (c) Controlling the scaffold degradation needs precise modification. If a scaffold degrades rapidly, mechanical failure may occur. Conversely, if a scaffold degrades slowly, an inflammatory reaction may be activated, hindering tissue repair. Hence, the balance between seasonable degradation and new bone formation is imperative and has proved a significant challenge,^290^ (d) Although current 3D construction techniques can be used for the construction of individualized patient defect models, 3D‐printed bioceramics have challenges, including the brittleness, unsuitability for load‐bearing clinical treatment, and potential harmful effects of toxic solvents and high temperatures on cell viability.^291^, ^292^, ^293^ In addition, the current 3D bioprinters lack the capacity of describing internal pore architecture, requiring further optimization for clinical translation,^294^ (e) The improvement of scaffold mechanical features has been discussed above. The individual tailoring of mechanical performance is not sufficient and satisfactory mechanical performance requires attention to a series of factors in scaffold construction, including the modification of compressive, tensile, elastic, and fatigue resistance. Combining these factors with other properties may be conducive to help stimulate osteogenesis,^295^, ^296^, ^297^ (f) Large‐scale defects and potentially high numbers of patients need to be treated by increasing the rate of biofabrication and scaffold production.^298^, ^299^ Additive manufacturing with high‐resolution bioprinting approaches only offer smaller‐scale manufacturing solutions, for example, the SLS‐based system has not been applied in the biomedical field for mass production due to the high cost. Nevertheless, in view of the commercialization and standardization of future raw materials, the realization of large‐scale production through SLA‐based and FDM‐based system will arrive in the near future.

## 9 CONCLUSIONS AND FUTURE DIRECTIONS

As described above, considerable efforts have been exerted for the reconstruction of bone tissue via biomimetic methods covering several biomimetic acellular strategies. To date, these advances, combining structural design, surface modification, and the application of external physical stimuli have huge potential for bone tissue regeneration. However, significant challenges remain in mimicking the structure and features of bone tissue as well as in the broad application of acellular materials to facilitate tissue repair. It is known that biochemical cues (e.g., growth factors, hormones, and chemokines) can regulate biological responses in the human body, yet their side effects, high cost, and lability hamper the translation into clinical applications. Recent developments demonstrated that bone regeneration in vivo may be achieved through the combination of internal structural cues and external physical stimulation, thus decreasing the dependence on exogenous cells and biochemical cues in bone tissue engineering. Furthermore, the resulting scaffold‐based bone tissue engineering treatments which are safe, convenient, and, importantly, cost‐effective, offer significant advantages for their clinical application.

As for the future outlook for ion‐functionalized materials, compared to single ion applications for bone regeneration, multielemental composites have been considered to be able to further enhance bone regeneration, or even synergistically improve bone repair; for example, silver is used for its antimicrobial activity and other ions for the promotion of osteogenesis and angiogenesis. Another example is the demonstrated synergistic effects of Co^2+^ and Mg^2+^ with HA on osteogenesis and angiogenesis.^300^ In the future, more attention should be paid to these multielement applications including the use of graded materials that can release ions sequentially. Another major concern is the precise control of the kinetics of ion release. With further research, ion‐functionalized materials will show great potential for bone tissue regeneration.^31^

The future prospects in decellularized tissue engineering should be considered. If biomaterials can perfectly mimic the structural and functional properties of the native tissue ECM, including encapsulation of the cell, stimulation of cell growth and ECM production, they may represent ideal scaffolds for tissue repair in the field of tissue engineering.^301^ Although synthetic materials have their benefits, such as tunable physical and chemical properties, they are unable to fully replicate the functions and structures of the native tissue, even with modifications or the addition of bioactive factors.^302^ Hence, the use of dECM in tissue engineering will create an environment that is able to mimic the characteristics of native tissue and to repair the injury site. However, limitations of the application of dECM in standard clinical therapies still exist. Various methods for decellularization have been reported, making it difficult to make decisions on which method is best for a specific application. Keeping the balance between eliminating fully cellular components so as not to trigger an immune reaction and trying to preserve the ECM composition to maintain biological activity is a great challenge that needs further research.^303^ Additionally, improving the methods to enhance the physical and chemical properties of the dECM while utilizing its inherent regenerative capacities will be the key to offer feasible treatment schemes for bone tissue regeneration.

The promise of tunable morphological and structural properties of certain materials has great potential. As described above, the complex micro‐ and nano‐scale architectures present in native bone tissue have been utilized many times in tissue engineering. Future work is required to address the intricacies of these features in 3D spatial modeling. It appears that the nanostructured surfaces (nanosheet and nanorod), rather than the release of Si ions, contribute most to the early cellular response while Si ions are mainly responsible for the promotion of BMSC differentiation.^87^ Thus, it is essential to systematically investigate this cooperation between Si‐substitution and nanosurface structures. The HAp convex micropatterned bioceramics demonstrated by Zhao et al.^88^ here might enhance osteoinductive ability through direct contact with both cells and the surrounding tissue. In addition, these micropatterned HAp bioceramics may provide a basis for the future development of bone implant scaffold materials as superior substrates, while the effects of the micropatterns on BMSC adhesion, proliferation and differentiation require further investigation. Smart, elastic, highly versatile, and growth factor‐ and cell‐free 3D biomimetic scaffolds with, above all, self‐fitting capability are the ideal features of materials for tissue regeneration, especially for bone defect repair.

The future direction of the physical cues on stimuli‐reactive scaffolds needs to be guided correctly. The significant potential of photothermally, electrically, magnetically, and mechanically responsive scaffolds in bone tissue engineering has been suggested. In terms of the safety and effects of the stimuli in the human body, relatively few in vivo and clinical studies have been reported. Thus, the promotion of the cell‐ECM interaction is in its infancy and future directions aim to determine the underlying mechanisms of mechanotransduction in the mechanical stimulation of cells and the role of external cues such as substrate stiffness. Additionally, the synergistic effect on osteogenic differentiation with BMP‐2 signaling and mechanotransduction has been investigated, based on BMP‐2 cues and different degrees of substrate stiffness. Hence, the combination of mechanobiological and biochemical phenomena will be the novel direction in the field of future bone tissue regeneration.^304^

**FIGURE 18:**
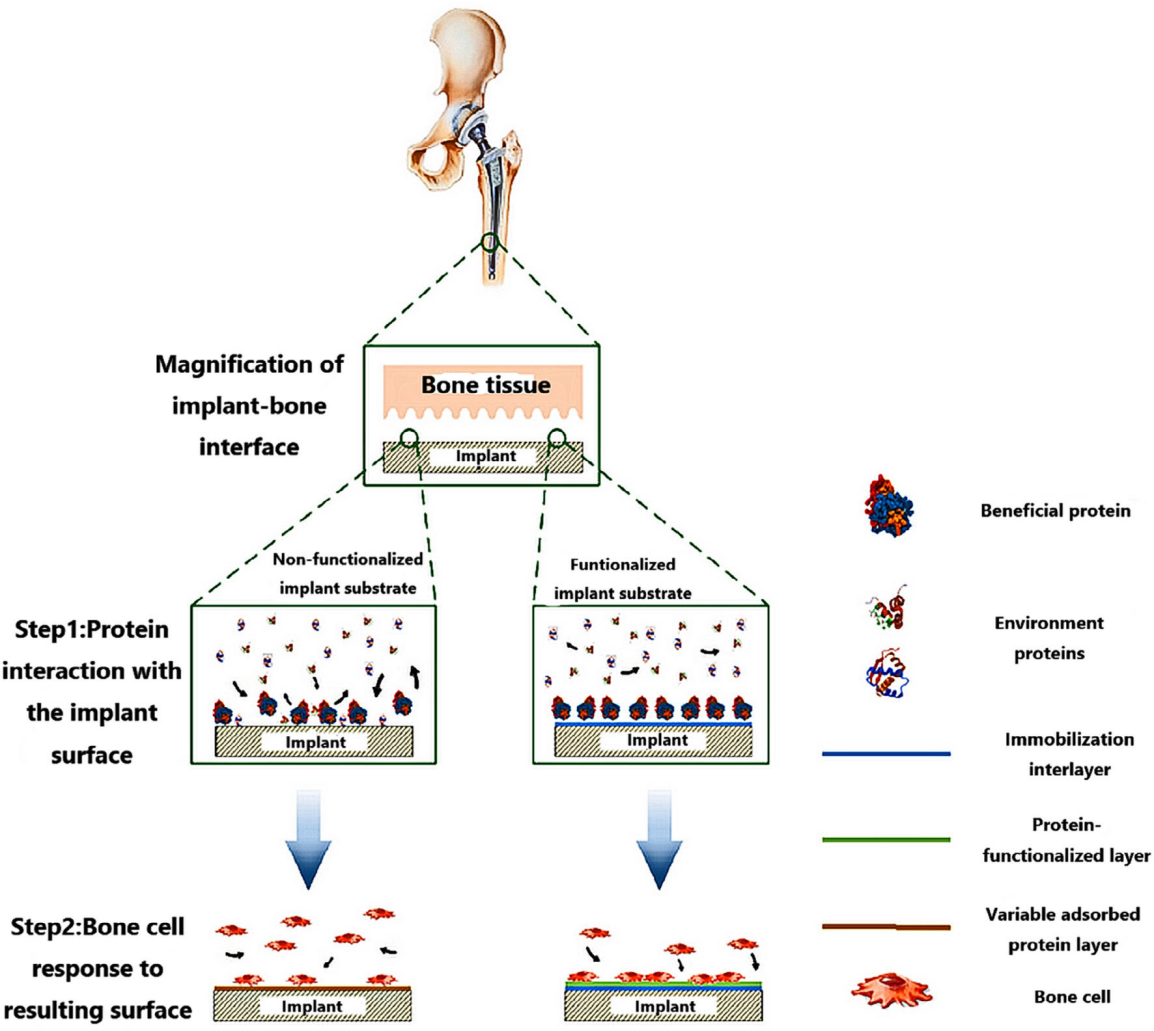
A diagrammatic representation of the initial stages of implant osseointegration for nonfunctionalized (current orthopedic implants) and biomolecule‐functionalized implants. After implantation, the implant surface interacts with the biological environment. The surfaces of nonfunctionalized implants become coated in proteins from the environment forming a variable protein layer (as described by the Vroman effect). The cellular response to the variable protein layer changes with the composition leading to unfavorable immune response, infection, or failure to integrate. Biomolecule‐functionalized surfaces produce a defined layer of biomolecules for a more controlled biological response leading to improved osseointegration *Source*: Adapted with permission (Stewart et al.^173^)

As interdiscipline of materials and medicine, the materdicine should draw more our attention to the underlying mechanisms of the interactions between the grafted scaffold materials and the microenvironment of the bone defect areas.^305^ The biological functionalization of material surfaces, due to its superiority on the improvement of biofunction, has been widely investigated. For example, the protein functionalization which requires the specific active sequence of the biomolecules in the microenvironment can attract resident endogenous cells and allow them to adhere gradually to the surfaces,^173^ indicating the critical importance of incorporating these biomolecules into the design of biomaterials and scaffolds for bone repair (Figure 18).Furthermore, sequestration of innate proteins such as growth factors by biomaterial‐based approaches is conducive to avoid the requirement of exogenous administration and has more potential in regenerative medicine.^306^ In comparison to other publications resembling this review article,^14^, ^31^ we comprehensively summarize the material and design of bone‐mimicking scaffolds, their production techniques, and the current strategies for clinical bone repair, as well as techniques to facilitate bone regeneration through modification of the characteristics of scaffold materials. Scaffolds have been constructed using acellular approaches including ion‐functionalization, incorporation of dECM, micro/nano‐features, physical stimuli, and immobilization of active factors, as well as “smart” features, indicating the advances and prospects of engineered bone tissue scaffold materials.

All in all, bone tissue engineering will continually advance toward the way of innovation of materials and scaffold designs inspired by the biomimicry of the hierarchical structures of bone tissue, as well as further investigation of underlying mechanisms in vivo for the promotion of bone regeneration, leading to the final realization of successful clinical translation.

## AUTHOR CONTRIBUTIONS

**Sijing Jiang:** Conceptualization; data curation; investigation; methodology; resources; writing‐original draft; writing‐review and editing. **Mohan Wang:** Conceptualization; data curation; formal analysis; resources; writing‐original draft; writing‐review and editing. **Jiacai He:** Conceptualization; data curation; formal analysis; funding acquisition; resources; writing‐original draft; writing‐review and editing.

## CONFLICT OF INTEREST

The authors declare that there is no conflict of interest that could be perceived as prejudicing the impartiality of the research reported.

## Data Availability

The data supporting this systematic review are from previously reported studies and datasets, which have been cited. Data sharing is not applicable to this article as no new data were created or analyzed in this study.
